# Single-trial dynamics of motor cortex and their applications to brain-machine interfaces

**DOI:** 10.1038/ncomms8759

**Published:** 2015-07-29

**Authors:** Jonathan C. Kao, Paul Nuyujukian, Stephen I. Ryu, Mark M. Churchland, John P. Cunningham, Krishna V. Shenoy

**Affiliations:** 1 Electrical Engineering Department, Stanford University, Stanford, California 94305, USA; 2 Bioengineering Department, Stanford University, Stanford, California 94305, USA; 3 School of Medicine, Stanford University, Stanford, California 94305, USA; 4 Palo Alto Medical Foundation, Palo Alto, California 94301, USA; 5 Department of Neuroscience, Columbia University, New York, New York 10032, USA; 6 Department of Statistics, Columbia University, New York, New York 10027, USA; 7 Neurosciences Program, Stanford University, Stanford, California, USA; 8 Neurobiology Department, Stanford University, Stanford, California 94305, USA; 9 Bio-X Program, Stanford University, Stanford, California 94305, USA; 10 Stanford Neurosciences Institute, Stanford University, Stanford, California 94305, USA

## Abstract

Increasing evidence suggests that neural population responses have their own internal drive, or dynamics, that describe how the neural population evolves through time. An important prediction of neural dynamical models is that previously observed neural activity is informative of noisy yet-to-be-observed activity on single-trials, and may thus have a denoising effect. To investigate this prediction, we built and characterized dynamical models of single-trial motor cortical activity. We find these models capture salient dynamical features of the neural population and are informative of future neural activity on single trials. To assess how neural dynamics may beneficially denoise single-trial neural activity, we incorporate neural dynamics into a brain–machine interface (BMI). In online experiments, we find that a neural dynamical BMI achieves substantially higher performance than its non-dynamical counterpart. These results provide evidence that neural dynamics beneficially inform the temporal evolution of neural activity on single trials and may directly impact the performance of BMIs.

## Abstract (web summary)

In online experiments with monkeys the authors demonstrate, for the first time, that incorporating neural dynamics substantially improves brain–machine interface performance. This result is consistent with a framework hypothesizing that motor cortex is a dynamical machine that generates movement.

## Graphical abstract

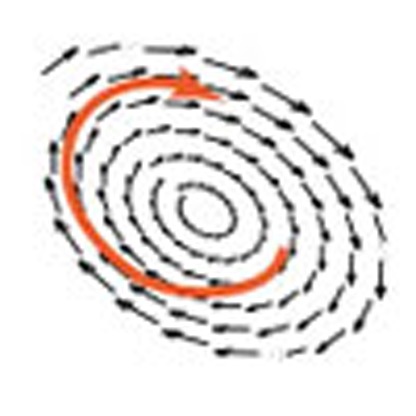

Consider the problem of estimating the trajectory of a cannonball fired at dusk. At your disposal is a low-resolution video camera that has only a few hundred pixels with poor sensitivity to incoming photons. A naïve approach is to take a video of the cannonball's flight and trace out its trajectory frame-by-frame. However, the resultant trajectory would be very noisy due to both the limited resolution of the camera and Poisson noise in photon detection (Fig. 1a–c). In this case, the observations are corrupted by noisy events that worsen trajectory estimation. This ‘observations only' based approach, however, can be substantially improved by incorporating knowledge of Newtonian mechanics. The cannonball has mass and is subject to physical laws that dictate its trajectory, such as the laws of motion, gravity and air resistance. These laws can be encapsulated in a linear dynamical system, where the cannonball's state (such as its position, velocity and acceleration), **z**_*k*_, can be inferred from its state a time step earlier, that is, **z**_*k*_=**Fz**_*k*−1_. By leveraging knowledge of the dynamical rules the cannonball obeys, we can augment the noisy video camera observations to obtain a better estimate of the cannonball's trajectory (Fig. 1d) that more closely matches the true flight of the cannonball.

In motor neuroscience, we have similar observation limitations. Our neural observations are both low-resolution (on the order of hundreds of electrodes) and noisy (with the arrival of action potentials being Poisson-like). However, a recent body of literature hypothesizes that analogous dynamical laws, describing how the activity of population of neurons evolves through time, exist in motor cortex^1^,^2^,^3^,^4^,^5^,^6^,^7^. These dynamics characterize how the neural population activity modulates itself over time (for example, through recurrent connectivity^8^,^9^) so that the neural population activity at time *k* is informative of the population activity at time *k*+1. Neural dynamical models of motor cortex are descriptive tools of these dynamics and typically posit that the spiking activity of a population of neurons arises from a latent (unobserved) neural state^3^,^5^,^10^,^11^,^12^. This neural state captures the shared variability in the neural activity, and as such summarizes the activity of the population^1^,^2^,^9^,^10^,^13^,^14^,^15^,^16^,^17^,^18^,^19^,^20^,^21^. Because the activity of the population is correlated, the dimensionality of the neural state required to capture a substantial proportion of the neural variance tends to be smaller than the number of cells recorded. In simple reaching tasks, this dimensionality has been observed to be 10–20 dimensions^10^. These latent state models have been effective in modelling the correlated behaviour of a neural population^10^,^11^,^12^ as well as in predicting behavioural correlates, including reaction time^22^.

An important prediction of neural dynamical models is that neural population activity observed up to the current time is informative of neural activity that has yet-to-be-observed on noisy single-trials. This concept, analogous to the cannonball example, is pictorially illustrated in Fig. 1e. In this hypothetical example, the neural state moves with purely rotational dynamics. A neural state estimated from the observed neural population activity alone without dynamics (for example, as with principal component analysis, PCA, or factor analysis) is very noisy on single trials. This is illustrated by the blue trajectory in Fig. 1e. However, if we had knowledge that the neural trajectories obeyed certain dynamics, as illustrated by the flow fields depicted in Fig. 1e, then the dynamics could be used to compute an *a priori* estimate of the next neural state (purple arrows in Fig. 1f). The subsequent neural observation would then update this *a priori* state estimate, as shown by the blue arrows in Fig. 1f, to yield a dynamically estimated neural state, depicted by the orange trajectory. This resultant trajectory incorporates information from both the neural dynamics and the neural observations. Performing dynamical estimation may have beneficial smoothing and denoising properties; in our example, a neural state trajectory rotating counterclockwise should not instantaneously traverse a clockwise path, as might be observed due to single-trial noise, just as a falling cannonball should not defy gravity and float up. This dynamical estimation should result in more accurate neural state trajectories than could be inferred by merely smoothing the observations without knowledge of neural dynamics.

While previous studies have performed systems identification to characterize the neural dynamics in motor cortex^3^,^5^, we sought to see if neural dynamics were informative of future neural activity and could therefore aid in single-trial neural state estimation. As single-trials are very noisy, does a neural dynamical model provide a measurable benefit over merely assuming a local smoothness in the neural activity? Demonstrating a benefit in forward prediction on single trials is central for the dynamical systems framework. Thus, we not only performed further systems identification of dynamical laws on single-trials (by analogy, characterizing gravity and air resistance), but also investigated whether these dynamical laws could improve single-trial estimation by dynamically smoothing and denoising the neural state (by analogy, improving our estimate of the cannonball's flight by using physical laws to smooth and denoise the cannonball's kinematics). We built and characterized dynamical models of single-trial motor cortical activity and asked if these models captured salient features of the neural population responses and were informative of how neural population responses evolve on single trials. We note that a ‘true' neural state cannot be inferred from our noisy single-trial observations. Thus instead, we used closed-loop brain–machine interface (BMI) performance as an indicator of the quality of neural state estimation. Further, if dynamical neural state estimation improves closed-loop BMI performance, this may have significant implications on BMI design^23^.

## Results

To learn and evaluate dynamical models of motor cortical activity during reaching, we recorded neural activity while rhesus macaques performed a center-out-and-back reaching task with eight targets positioned on the circumference of a circle with 12-cm radius (Supplementary Fig. 1, Methods). The monkey was required to hold each target for 500 ms. Neural activity was recorded from implanted electrode arrays in dorsal premotor cortex (PMd) and primary motor cortex (M1). Monkey J had two 96-electrode Utah arrays, one implanted in PMd and one in M1, while Monkey L had one array implanted at the border of PMd and M1.

### Learning single-trial motor cortical dynamics

We modelled recorded spiking activity during reaching using an autonomous latent-state linear dynamical system (LDS). In the LDS, the observed neural population spike counts at time *k*, **y**_*k*_, can be interpreted as a noisy observation of a low dimensional and dynamical neural state, **s**_*k*_. In this work, **y**_*k*_ was the threshold crossing spike counts on each electrode in non-overlapping 15-ms bins. We chose the neural state to be 20-dimensional as to be sufficiently high enough to capture a substantial proportion of the neural variance^10^. We modelled this system in the linear Gaussian form:


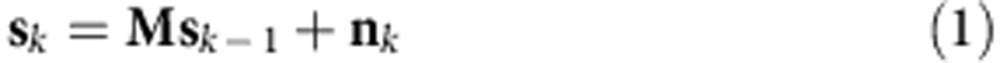



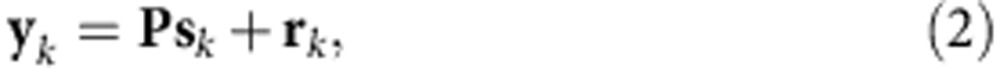


where **n**_*k*_ and **r**_*k*_ are zero-mean Gaussian noise terms. We refer to equation (1) as the dynamics process and equation (2) as the observation process. The LDS parameters were learned in an unconstrained fashion from neural population responses observed during 500 reaching trials (corresponding to ∼30,000 bins of data) with expectation maximization (Methods, Supplementary Fig. 2). This approach finds LDS parameters, which (locally) optimize the log-likelihood of the observations under the assumptions of equations (1) and (2) (ref. 24). We found that **M** always converged to a full-rank, non-normal matrix with unique and stable eigenvalues, as characterized in Supplementary Figs 3 and 4.

**Figure 1:**
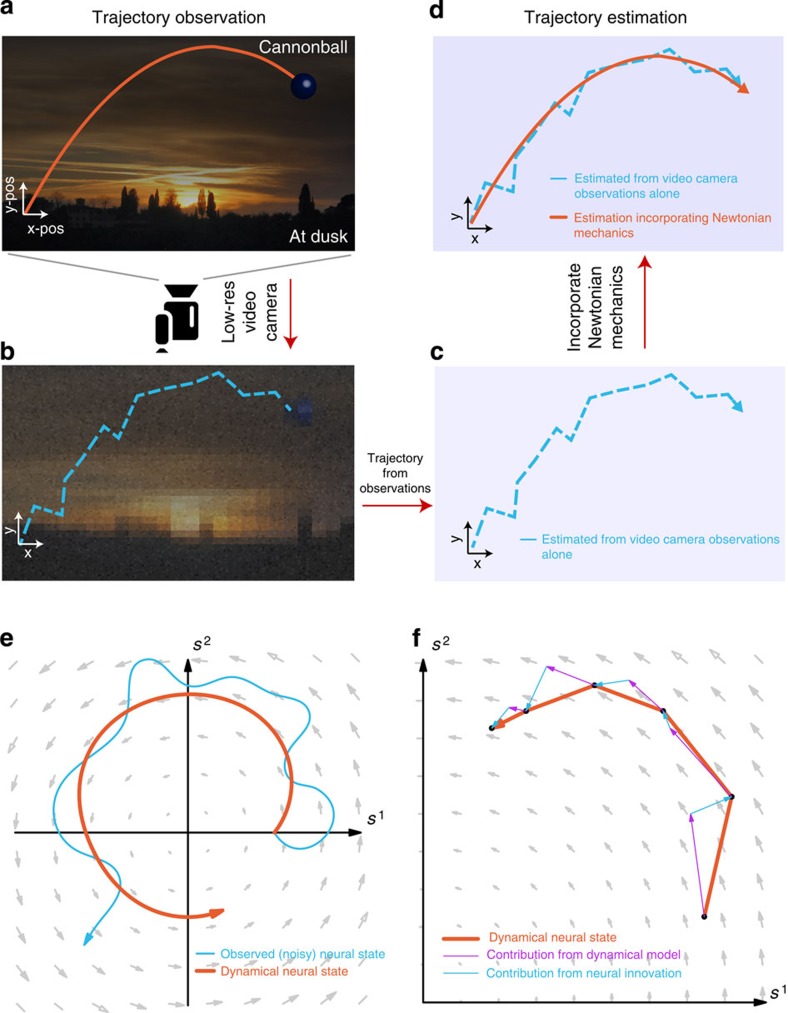
Incorporating dynamics into trajectory estimation. (**a**) An illustration of a cannonball being fired at dusk, following a parabolic trajectory (orange) according to physical dynamical laws. Photo courtesy Christine Wong. (**b,c**) The flight of the cannonball is recorded by a low-resolution video camera. However, two factors obscure the true path of the cannonball. First, the camera is low resolution, only being able to capture hundreds of pixels with poor colour resolution. Second, the incoming photons are characterized by Poisson noise, which in part corrupts the image. As a result, the trajectory path recorded in the video might appear very noisy, as illustrated by the dotted-blue trajectory. (**d**) By incorporating knowledge of Newtonian mechanics, **z**_*k*_=**Fz**_*k*−1_, we can arrive at a better estimate of the flight path of the ball. This estimate incorporates knowledge of the laws of motion, the theory of gravity, and air resistance. For example, the ball should not defy gravity by floating up while it is falling. (**e**) An illustrative analogue of the cannonball in a 2D projection of neural state space. In this toy example, the arrows indicate the dynamics of the neural state, so that for the purposes of this illustration, it rotates counterclockwise. When the dynamics are not taken into account, the neural state trajectory inferred only from noisy single-trial neural observations may be very noisy (blue trace). However, the neural state trajectory noise may be ameliorated by accounting for the neural state dynamics (orange trace). (**f**) One way in which dynamical information may be included is to linearly weigh the predicted neural state, as a result of a dynamical model and the neural ‘innovations', which is derived from the neural observations. The relative weight given to the dynamical process versus the observation process is influenced by factors such as their noise processes. The relative weight of the contribution from the dynamical process versus the neural innovations is reported in the Results.

Equations (1) and (2) describe how the previous neural state, **s**_*k*−1_, and the current neural observation, **y**_*k*_, are informative of the current neural state, **s**_*k*_. To estimate **s**_*k*_ given **s**_*k*−1_ and **y**_*k*_ we used the Kalman filter, which is a minimum mean-square error estimator of a Gaussian LDS. We denote the Kalman filter estimate of **s**_*k*_ as 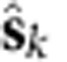. With the Kalman filter, the contribution of the dynamics process (using the previous neural state estimate, 
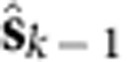
) versus the ‘innovations process' (using the neural observations, **y**_*k*_) in estimating the neural state can be computed by calculating the norms of the vectors in Fig. 1f (described further in the Methods). Across 13 experimental days in Monkey J and 16 days in Monkey L, where a new LDS was learned on each experimental day, we found that the dynamics process contributed 37±2% (mean±s.d.) and 49±4% to the state estimate. The consistency of this proportion across days suggests that LDS learning was fitting underlying structure in the neural population activity rather than simply fitting noise, as further supported by cross-validating the model likelihoods (Supplementary Fig. 2). Thus, for our parameters, linear dynamics explained 35–50% of how the neural population responses evolve through time. Further, we found that these contributions were consistent across experimental days within subject, but differed between subjects (due to different cell sampling). This suggests that there is consistent structure in how observed neural population responses evolve that can be robustly captured by a linear dynamics process. Moreover, we found that the single-trial neural dynamics had consistent eigenvalue characteristics that were qualitatively similar to previous reports in non-human primates^5^ and humans with ALS^7^ (Supplementary Figs 3 and 4).

**Figure 2:**
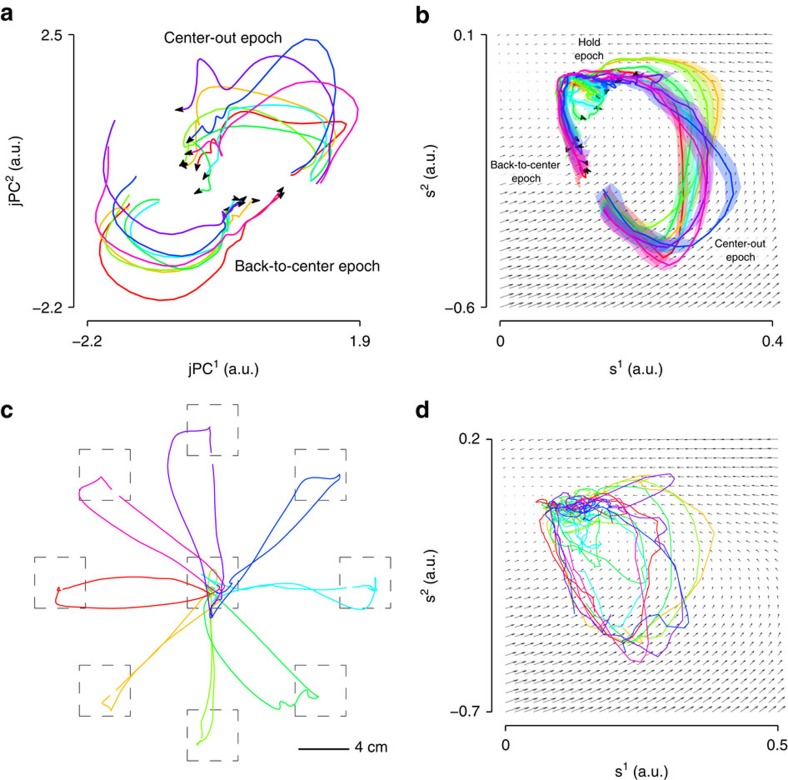
Trajectories of the neural state. (**a**) Trajectories of the condition-averaged neural data are shown using jPCA, which finds planes that capture rotational structure in the data. The jPCs are rotations of the principal components. (**b**) Trajectories of a dynamical neural state, inferred by a Kalman filter using cross-validation data, for center-out-and-back reaching. These trajectories are the averages of single trials (s.e.m. shown in shading). Also shown are the dynamics of the learned dynamical system. During the center-out epoch, the trajectories appear to follow the dynamical flow fields. Moreover, during the hold epoch, the strength of the flow field appears weaker. It is worth noting that because the dynamics shown are only 2-dimensional, they are far less rich than the 20-dimensional dynamics and may not adequately capture the dynamics of all portions of the reach (such as during the back-to-center epoch). (**c**) Behavioural kinematics (hand position) on single trials of Monkey J performing the center-out-and-back task (Methods). (**d**) The single-trial neural trajectories corresponding to the same single-trial reaches in **c**.

We emphasize that our estimate of the neural state incorporates a dynamics process in contrast to other dimensionality reduction techniques based on PCA^5^,^14^,^15^,^25^ and factor analysis^26^,^27^. For example, jPCA^5^,^7^ (trajectories shown in Fig. 2a) does not use a dynamics process, but rather finds a rotation of the principal components showing rotational structure in the neural population activity. In the cannonball example, this is analogous to systems identification (that is, characterizing the dynamical laws governing the movement of the cannonball), whereas we actively use the dynamics to infer a new trajectory (that is, denoising the observed trajectory of the cannonball). To visualize the neural state trajectories of an LDS, we learned a highly constrained LDS with a 2-dimensional (2D) (rather than a 20-dimensional) latent state, since it is infeasible to visualize dynamics of a 20-dimensional space. We stress that the 2D LDS is only shown for visualization purposes and is a very limited model because it does not capture a substantial proportion of the neural variance. Moreover, because these 2D dynamics are far less rich than those of a 20-dimensional system, it is prone to underfitting and may not fully model different portions of the reach where dynamics differ^14^,^15^. Nevertheless, as shown in Fig. 2b using cross-validation neural activity (where trajectories are condition-averaged across single trials), we observed that the neural state trajectories travelled along directions guided by the neural dynamics, as depicted by the flow fields, during the center-out and hold epochs of the reach. This was also the case on single trials, as shown in Fig. 2d (with the corresponding behavior shown in Fig. 2c).

### Neural dynamics are slower during holding than reaching

**Figure 3:**
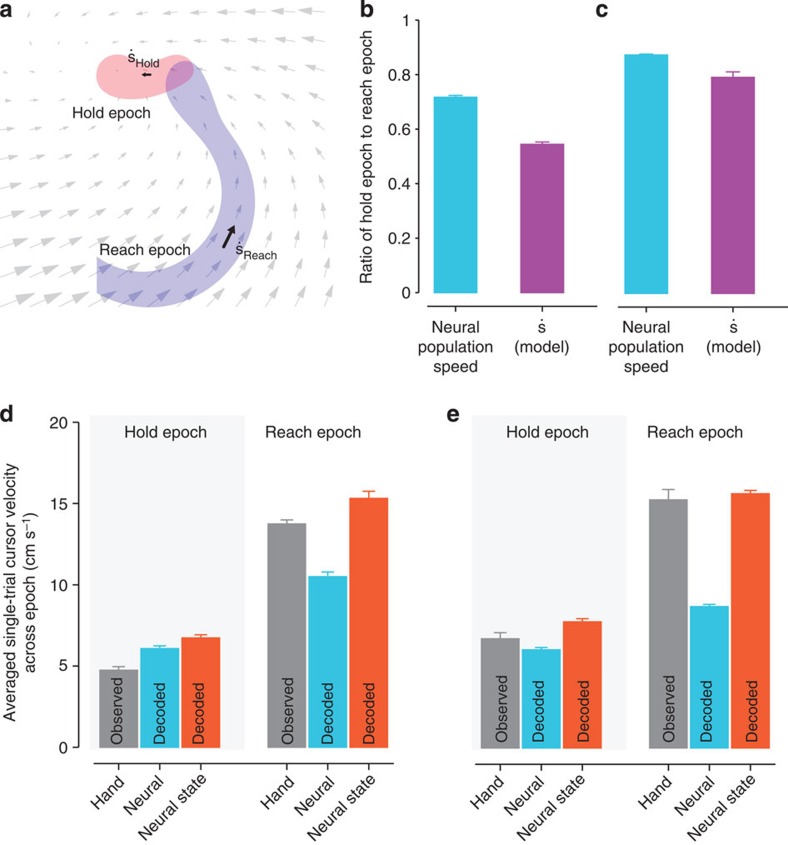
Neural dynamics accentuate the dynamic range of velocities in hold versus reach. Bar plots in this figure represent the average across seven experimental days in Monkey J and six experimental days in Monkey L, and the error bars denote the s.e.m. (**a**) A 2D illustration of neural state trajectory regions during the hold and reach epochs, as well as the dynamics they obey. The goal of this analysis is to assess the relationship between 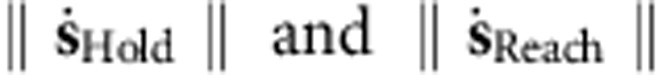. (**b**) The ratio of the (high-dimensional) neural population speed during the hold epoch to the reach epoch is 0.72 (blue), indicating that the rate of change of the neural population activity is less in the hold epoch than in the reach epoch. The dynamical model captures this feature, with the ratio of the neural state speed predicted by the model in the hold epoch to those in the reach epoch being 0.55 (purple). (**c**) Same as (**b**), but for Monkey L. The ratio of the high-dimensional neural velocities is 0.88; the ratio of the model-predicted neural state velocities is 0.79. (**d**) The averaged single-trial hand velocities during the hold and reach epochs are shown in grey. The average of the decoded single-trial velocities during the hold epoch are comparably low for both the high-dimensional neural data (**y**_*k*_, blue bar) and the dynamical neural state (**s**_*k*_, orange bar). However, during the reach epoch, the dynamical neural state is able to decode significantly higher velocities. (**e**) Same as (**d**) but for Monkey L.

In a dynamical system, the position of the state predicts the velocity of the state, as depicted by the flow fields in Figs 1e,f and 2b,d. Therefore, we asked: do the modelled neural dynamics capture salient features of the neural state velocity? Two distinct regions explored by the neural state are the region where the monkey is holding the target and the region where the monkey is reaching to the target, as illustrated in Fig. 3a. We first asked if the neural population speeds, given by the difference ||**y**_*k*+1_−**y**_k_||, are faster or slower during the hold epoch (from 100 ms after hold initiation to 150 ms before hold completion) versus the reach epoch (center-out trials). Across seven experimental sessions in Monkey J and six in Monkey L, we found that the ratio of the mean neural population speeds during the hold epoch to those during the reach epoch was on average 0.72 and 0.88 (Fig. 3b,c; blue bars). Thus, the neural population responses exhibit a smaller rate of change when the monkey is holding a target than reaching to a target. This may be a result of the monkey producing more static kinematics and electromyography when holding rather than reaching to a target.

We next assessed whether single-trial dynamical models predicted slower neural-state speeds during the hold epoch versus the reach epoch. We calculated the first-order model-predicted neural-state speed, ||**s**_*k*+1_−**s**_*k*_||=||(**M**–**I**)**s**_*k*_||, for all neural states in the hold epoch and in the reach epoch using cross-validation data. We found that the ratio of the mean model-predicted speeds between holding and reaching was 0.55 in Monkey J and 0.79 in Monkey L (Fig. 3b,c; purple bars). Therefore, the neural dynamics produced relatively smaller neural state velocities during holding than reaching. In this sense, the neural population responses during target holding reside in a region of slower dynamics of the neural state space. Incorporating neural dynamics into state estimation may therefore beneficially accentuate neural state velocity differences between holding and reaching on noisy single-trials. For example, if the neural dynamics denoise noisy observations by accelerating the neural state more during the reach epoch than during the hold epoch, we may be able to decode a more accurate dynamic range of hand velocities on single trials. We found this was the case (Fig. 3d,e and further described in Supplementary Table 1), as a decoder using the neural state (rather than the neural observations) to estimate cursor velocity was as quick as the hand during the reach epoch with appropriately low velocities during the hold epoch (Supplementary Table 1). The ability of a decoder to generate both quick and slow velocities is crucial for high-performance BMIs^28^,^29^.

### Neural dynamics as a predictor of future neural activity

Because the neural dynamics predict how the neural state evolves differently depending on its location state space, we next asked if neural dynamics predict yet-to-be-observed future neural population activity better than just assuming a local smoothness in the neural activity. We evaluated how much of the variance in the neural observations at time *k*, **y**_*k*_, could be explained given neural observations up to time *k*−1. Because observed spike counts are noisy at the single trial level, we performed predictions over ∼15,000 bins of neural observations on each of 13 different experimental days in Monkey J and 15 experimental days in Monkey L.

We first evaluated if using neural dynamics could better predict the neural population activity at time *k* than merely assuming that the neural activity is locally smooth and constant at a 15-ms bin resolution (with no dynamical predictive model). Therefore, we first calculated how much variance in **y**_*k*_ could be predicted by the causally smoothed neural observations at time *k*−1, given by 
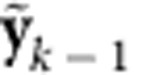
. The smoothing kernel was a causal Gaussian kernel with a s.d. of 100 ms. We found that the smoothed neural observations captured 22% and 25% of the future neural variance in Monkeys J and L, respectively. To predict future neural observations with the neural dynamical model, we used the single-trial dynamics to estimate the *a priori* neural state at time *k*. This estimate of the next neural state at time *k* was then used to estimate the neural activity at time *k* (via the observation process). Therefore, the estimator of **y**_*k*_ was 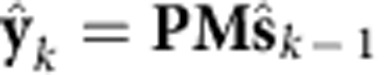. We found that the neural dynamical model captured 31% and 29% of the future neural variance. This represents an increase in the captured variance by 9 and 4%, a 43 and 16% (*P*<0.01, paired *t*-test, both monkeys) improvement over using the smoothed neural observations from 15 ms earlier.

Further, we asked: are single-trial neural dynamics better descriptors of future neural population responses than those learned from condition-averaged neural population responses^5^? If neural population responses obey dynamical laws, then these laws would operate on single trials. We learned LDS's from condition-averaged neural population responses and found that they captured 28 and 25% of the future neural variance in Monkeys J and L. Hence, the single-trial dynamics still increased the captured variance by 3 and 4%, which is an improvement of 10 and 16% over the condition-averaged LDS (*P*<0.01, paired *t*-test, both monkeys). This shows that single-trial dynamics better model the evolution of single-trial neural population responses than do condition-averaged dynamics, suggesting that learning dynamics on single-trials captures dynamical features that are obscured in the condition-averaged neural activity.

### Incorporating neural dynamics increases BMI performance

By using neural dynamical models to predict how the activity of a neural population evolves over time, can we arrive at a ‘better' and potentially denoised single-trial neural state? An obstacle in addressing this question is that a ‘true' neural state cannot be known from our observations. Therefore, we used an online BMI as an indicator of the quality of neural state estimation. If the dynamical model had good predictive power, we would expect the performance of the BMI to increase over one that did not incorporate a dynamical model. On the other hand, if the dynamical model had poor predictive power, we would expect the performance to decrease. We built a closed-loop BMI system where the neural dynamics were used to augment neural state estimation in a real-time feedback loop. We performed this study online (1) so that the subject could adjust his neural activity as a result of feedback of the cursor movements^28^,^30^, reflecting the newly and dynamically predicted neural state and (2) to assess the utility of incorporating neural dynamics into a clinical BMI.

**Figure 4:**
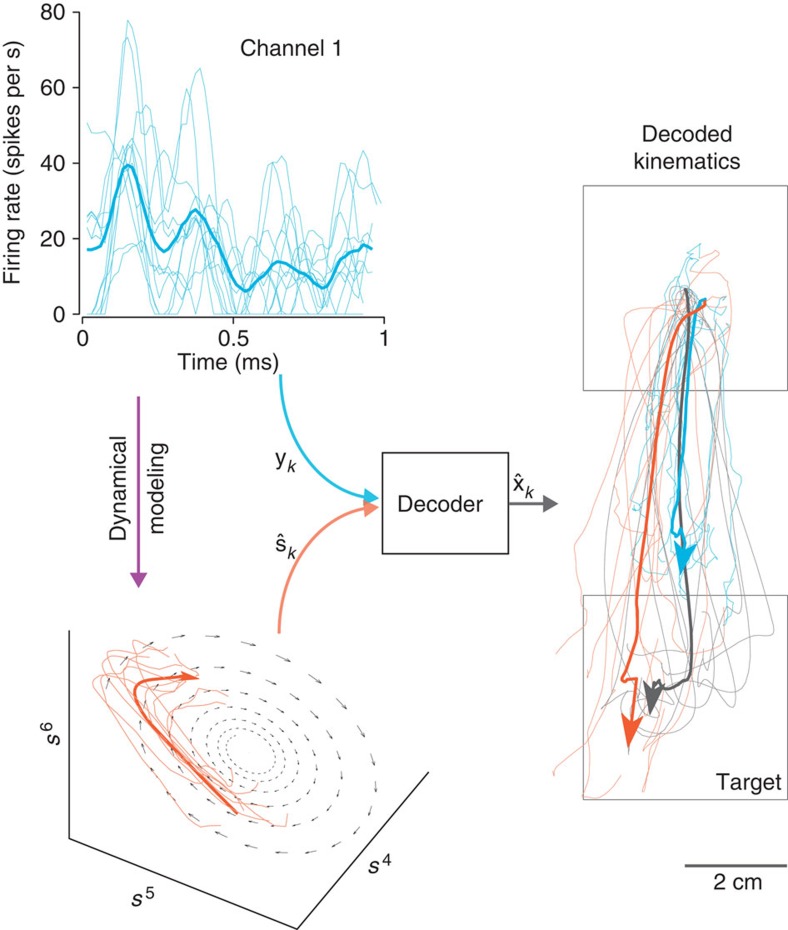
Decoding with a dynamical neural state as opposed to noisy neural observations. A decode algorithm takes neurally derived observations and outputs decoded kinematics, 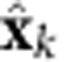. Most BMI systems decode using noisy neural observations, **y**_*k*_, which comprise the single-trial spike counts of the neural data. This data (smoothed) is shown for a single channel, where the light blue traces denote the single-trial neural observations when a monkey intended to reach downwards. The bolded trace is the firing rate averaged over trials (relative to the start of the trial), or peristimulus time histogram. To the right of the decoder block, the black traces denote the true path of the cursor for trials where the monkey reaches from the upper square target to the lower square target (where both squares are 4 × 4 cm). The bolded trajectory is the average path of the cursor across single-trials. The offline reconstructions using the dynamical neural state result in a superior offline decode when compared with using the the non-dynamical binned spike counts.

The concept of our experiment is illustrated in Fig. 4, where the same decode algorithm can be driven by either the noisy single-trial neural spike counts (blue traces) or by the dynamical neural state (orange traces). The output of the decoder is cursor kinematics (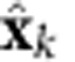). As suggested by the offline analysis of Fig. 4, the single-trial trajectories decoded by the neural state (thin orange lines) better replicate the true hand trajectories (black) than those decoded by the high-dimensional neural data (blue). To avoid obfuscation of results by using more complex decoder models, we decoded using simple least-squares regression. Therefore, we found least-squares optimal (**L**_*s*_, **b**_*s*_) and (**L**_*y*_, **b**_*y*_) to decode kinematics according to the following equations:


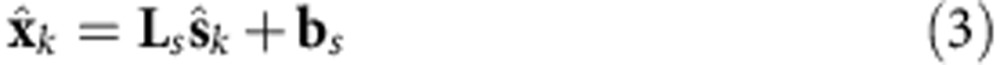



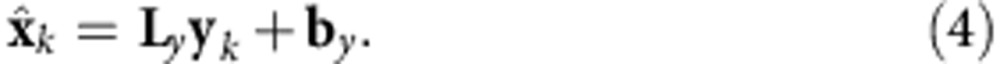


**Figure 5:**
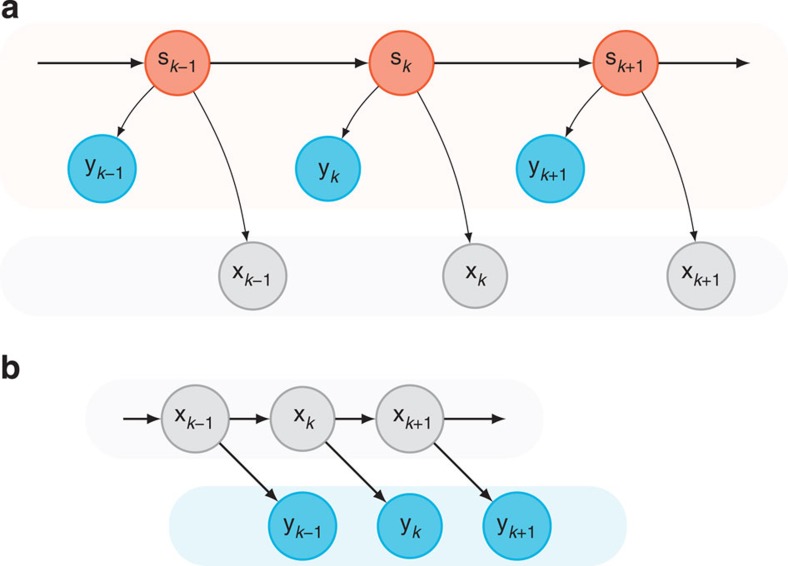
Graphical representation of decoder algorithms. (**a**) A graphical representation of a proposed neural dynamical filter, modelling the dynamics of the neural state (**s**_*k*_). The neural state propagates through time obeying modelled dynamics, at each point in time generating both the kinematics (**x**_*k*_) and the observed neural data (**y**_*k*_). (**b**) Graphical representation of the linear dynamical system underlying kinematic-state Kalman filters, where the kinematics (**x**_*k*_) are related through a linear dynamical update rule, and are causal to neural observations (**y**_*k*_). There is no temporal structure modelled in the neural activity. We also note that this model is of opposite causality to that of **a**, since kinematics are generative of the neural activity rather than neural population activity (reflected by the neural state) being generative of kinematics.

We refer to equation (3) as the neural dynamical filter (NDF), while equation (4) is the optimal linear estimator (OLE)^31^. A graphical representation of the NDF is shown in Fig. 5a, where the neural state propagates with dynamics, at each point generating the observed neural activity and kinematics. We note that despite the NDF having more parameters than the OLE, it is not trivial that the NDF would outperform the OLE in closed-loop experiments, where the monkey was incorporated in a feedback loop^30^,^32^,^33^. For example, if the learned dynamics did not describe the temporal evolution of the population activity well, it may be that merely smoothing the neural responses with a Gaussian kernel and finding a least-squares optimal mapping to the kinematics would result in similar or better generalization to online control.

**Figure 6:**
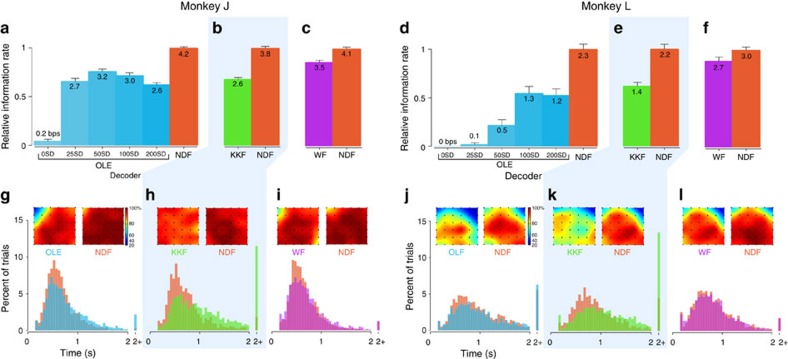
Online experimental performance of various decoders. We calculated the mean bitrates (error bars denote s.e.m. across experimental blocks) normalized by the bitrate of the highest performing decoder. More details on statistics and how we calculated mean bitrates are in the Methods and Supplementary Tables 2 and 3. (**a**) On the *x* axis, ‘X SD' refers to an optimal linear estimator (OLE) decoder where the neural data were smoothed by a causal Gaussian kernel with a s.d. of *X*  ms. The bitrate of the neural dynamical filter (NDF) is 31% higher than the best OLE with Gaussian smoothing (*P*<0.01, paired *t*-test). The absolute performance of each decoder is denoted by the text in each bar. The performance was evaluated across 29 experimental blocks (except for OLE000 that was evaluated on 25 of the 29 experimental blocks due to monkey motivation). (**b**) The NDF achieved a bitrate 47% higher than the kinematic-state Kalman filter (KKF; *P*<0.01, paired *t*-test). Differences in performance of the NDF across decoder comparisons can be attributed to monkey motivation as well as performance variability across weeks. For example, when a decoder performs poorly, such as the KKF, the performance of the NDF in these paired data sets tend to be lower. The performance was evaluated across 22 experimental blocks. (**c**) The NDF achieved a bitrate 16% higher than the WF (*P*<0.01, paired *t*-test). The performance was evaluated across 21 experimental blocks. (**d**) The NDF achieved a bitrate 83% higher than the best OLE with Gaussian smoothing (*P*<0.01, paired *t*-test). The performance was evaluated across 20 experimental blocks (except for OLE000 and OLE050 that were evaluated on 6 and 17 of the 20 experimental blocks, respectively). (**e**) The NDF achieved a bitrate 61% higher than the KKF (*P*<0.01, paired *t*-test). The performance was evaluated across 18 experimental blocks. (**f**) The NDF achieved a bitrate 13% higher than the WF (*P*<0.01, paired *t*-test). The performance was evaluated across 21 experimental blocks. (**g**–**i**) For Monkey J, the histograms of target acquire times for each decoder is shown (mean data in Supplementary Table 2). In the inset, the physical workspace of a 6 × 6 grid of targets is shown, and the target acquisition success rate in the workspace is represented by a heat map. (**j**–**l**) Same as g–i but for Monkey L.

We compared the performance of the NDF and OLE over 13 online experimental sessions in closed-loop BMI control on a generalization grid task that allowed for the computation of an achieved bitrate^34^,^35^ (Methods). We emphasize that this task evaluates the generalization of neural dynamics learned from center-out-and-back reaching to a scenario where targets are randomly selected from a grid of 36 targets, sampling a much more diverse set of conditions. Because the dynamics provide a measure of smoothness to the dynamical neural state, we allowed the neural spike counts to also be smoothed by convolution with causal Gaussian kernels having s.d. ranging from 25 to 200 ms. As shown in Fig. 6a,d, a BMI incorporating neural dynamics achieved significantly higher performance (as measured by achieved bitrate^34^,^35^ described in the Methods) than its non-dynamical counterpart. The NDF achieved 31% and 83% higher performance than the best OLE decoder in Monkeys J and L, respectively (*P*<0.01, paired *t*-test, bitrates estimated from 16,245 total online trials for Monkey J and 8,572 trials for Monkey L, breakdown in Supplementary Tables 2 and 3). We also found that the NDF achieved higher success rates than the OLE, as shown in Fig. 6g,j. A video of the performance of the NDF on the grid task is shown in Supplementary Movie 1, and a table of mean statistics can be found in Supplementary Tables 2 and 3. We also performed a control to demonstrate that this performance benefit was not solely a result of using smooth, low-dimensional components. We performed PCA and selected the first 20 components (equalizing the dimensionality of the LDS neural state) and effectively smoothed each component in time with a causal Gaussian kernel with s.d. 100 ms (‘PC-smooth'). We found that the performance of a decoder driven by the smooth PCs was still worse than the performance of the NDF (Supplementary Fig. 5). These results suggest that the performance benefit of the NDF is not merely due to its low-pass filtering properties, but rather may be a result of learning underlying structure in the data.

To what extent does the NDF capture neural population response structure as opposed to merely achieving high performance by smoothness and regularization attributed to the Kalman filter? We addressed this question with three additional experiments. First, we implemented a control where we relearned new decoders with a perturbed dynamics matrix; second, we compared NDF performance with a kinematic-state Kalman filter^23^,^28^,^36^,^37^,^38^ (KKF); third, we compared the NDF performance with a more general regularized linear model, the Wiener filter (WF).

In the first control, we permuted the columns of the dynamics matrix, **M**, and learned a new decoder with the perturbed dynamics. We observed that the performance of the decoder substantially declined, sometimes to the point where the monkey would not perform the task out of frustration. This demonstrates that when the dynamics do not model the evolution of the population responses well, performance substantially decreases.

Second, we predicted that the NDF should outperform a KKF^23^,^28^,^36^,^37^,^38^, which has a far simpler dynamical model that does not capture the richness of the neural population response dynamics. The KKF effectively smooths neural data by use of a dynamical model learned from the kinematic (cursor movement) data, in stark contrast to smoothing neural data by use of a dynamical model learned from the neural population, as proposed here. These kinematic dynamical update rules are characterized by exponential decay on the velocity^23^,^26^ (Supplementary Fig. 6). Over six experimental sessions, we found that the NDF performed substantially better than the KKF (47% and 61% improvement in Monkeys J and L, respectively, *P*<0.01, paired *t*-test, bitrates estimated from 4,500 total online trials for Monkey J and 3,683 trials for Monkey L) as shown in Fig. 6b,e. We also found that the NDF achieved significantly higher success rates and quicker acquire times^28^ than the KKF, as shown in Fig. 6h,k and Supplementary Tables 2 and 3. These results suggest that the neural dynamics capture structure in the neural activity that cannot be described by relatively simpler kinematic dynamical update rules alone.

Third, we compared an NDF with a more general linear model, the WF. The WF finds the optimal linear least-squares coefficients, **L**_0_, **L**_1_,…, **L**_*p*−1_ to decode the current kinematics as a function of a history of neural data, so that 
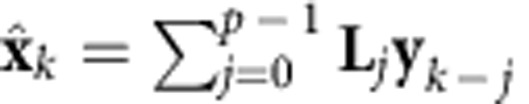
 as further described in the Methods section. Any linear state estimation in a dynamical system can be written as a linear operation on the history of the observed data. In this sense, the WF represents the most general model of any linear approach in that OLE, PC-smooth, KKF and NDF can be written in this form^23^,^39^. Even so, this does not guarantee that the WF will have superior generalization performance to other decoders, especially in online control where the statistics of neural activity differ from those during open-loop reaching because the monkey must compensate for errors made during decoding. We optimized the parameters of the WF (including the amount of history used, as well as the amount of regularization) in closed-loop experiments. We observed that the WF achieved higher bitrates in closed-loop control than the OLE and KKF. However, we found that the NDF performed significantly better than the WF (16% and 13% improvement in Monkeys J and L, respectively, *P*<0.01, paired *t*-test, bitrates estimated from 4,561 total online trials for Monkey J and 4,296 trials for Monkey L) as shown in Fig. 6c,f, and acquired targets at higher success rates, as shown in Fig. 6i,l and Supplementary Tables 2 and 3. Thus, even with the limitation that the modelled neural dynamics are linear, we found that directly modelling the neural dynamics resulted in performance that could not be matched by brute force linear regression. This suggests that modelling neural dynamics captures properties of the neural population that are not extracted by least-squares regression over a history of the neural data, even though the approach could in principle capture neural dynamics. Altogether, these results demonstrate that incorporating neural dynamical modelling into a BMI algorithm can substantially increase its performance and provide evidence that neural dynamics augment neural state estimation to arrive at a better single-trial estimate of the neural state.

## Discussion

If neural population responses evolve according to rules that are well captured by a dynamical system, then these dynamics should be capable of being ‘put to use.' That is, dynamical information should help augment single-trial neural state trajectory estimation. Putting dynamics to use entails both systems identification (learning a model of the dynamics of single-trial neural population responses implemented in motor cortex) and a demonstration that these dynamics aid in denoising noisy single-trial observations (as in BMIs where decodes occur on noisy single trials).

We characterized single-trial dynamics learned from motor cortical neural populations during reaching. Across experimental days, we observed a consistency in the modelled neural dynamics in terms of (1) the contribution of the dynamics process to neural state estimation, (2) the eigenvalue spectrum of the learned models, and (3) the ratio of model-predicted neural state speeds between holding and reaching. This suggests that the dynamical features we observe are not spurious or merely fitting noise, especially considering that such consistency was not guaranteed with our learning technique, which is subject to local optima. We note that, for our linear approximation of the dynamics, the single-trial neural dynamics were characterized by substantial exponential decay (Supplementary Figs 3 and 4). We found that to optimally estimate the neural state (in a mean-square error sense) the dynamics process contributed 35–50% in magnitude, which is not insignificant. Because these contributions are a function of the noise in the dynamics and observation processes, as well as other parameters, a better model of the dynamics process (for example, a nonlinear dynamics process^3^) may result in a larger contribution from the dynamics process.

We found that single-trial dynamics were more predictive of future neural activity than simply assuming a local smoothness in binned spike counts. One reason neural dynamics may improve forward prediction is because the neural dynamics capture distinct characteristics of the neural population responses in different regions of neural state space. For example, during the hold epoch of the reach, the neural state resides in a region with slower dynamics which decreases the neural state velocity. An important line of future work will be necessary to understand how networks of neurons may implement such dynamics in biologically plausible ways, and how empirically observed dynamics may constrain network architectures. Recent studies suggest that these type of neural dynamics may arise in recurrently connected networks of neurons^8^,^9^.

To provide evidence that these dynamics aid in neural state estimation, we used a BMI system as an indicator of the quality of neural state estimation. Performing this study in a closed-loop experiment was important because the dynamical neural state estimate will be reflected, to an extent, by the movements of the cursor, so that the subject could adjust his neural activity and make online feedback-based corrections. (In contrast, we note two related offline BMI studies by Wu *et al*.^40^ and Truccolo *et al*.^41^, further discussed in the Methods.) We observed that the online BMI controlled by the neural state achieved substantially higher performance than its non-dynamical counterpart, suggesting that these dynamics are aiding in neural state estimation in a beneficial way. In contrast, when we altered the dynamics process, or used a dynamical update rule learned only from the kinematics, we observed a substantial decrease in performance.

An interpretation from the point of view that motor cortex represents kinematic variables is that the dynamics observed in motor cortex may reflect the dynamical update rules of kinematic intent. In this sense, the graphical model of Fig. 5b (KKF) can be interpreted to demonstrate the progression of an ‘intended kinematic variable'^42^. Our results that an NDF outperforms a KKF suggest that this is not the case, and that the dynamics of neural population responses in motor cortex are far richer than those of kinematic intent. This further supports evidence that complex, heterogeneous motor cortical responses may have their own internal drive, as opposed to solely encoding external kinematic variables^43^,^44^.

We note that in our study, the monkeys were free to move the contralateral arm during BMI experiments. This is because we characterized the dynamics of reaching, and so the BMI should be controlled in a similar fashion. A subject who is constrained to not move may not fully explore the neural state space, having cortical activity that resides in a null space^15^ where neural dynamics may be substantially different than those during reaching. While we believe the monkey model where the subject is free to move his arm more closely resembles that of a paralysed patient than when both arms are restrained^34^,^45^, we note that future work should investigate the characteristics of the dynamics of imagined movements. For example, we may find that the dynamics of imagined movements may be more nonlinear, and require different dynamic modelling assumptions^3^,^9^,^46^,^47^.

The neural dynamical viewpoint has implications on the design of BMIs, including how to better smooth neural activity through time. Two standard techniques in BMIs, OLE and KKFs, smooth the neural data in a manner that does not use any knowledge of the neural data. Often times, OLE (or population vectors) will be accompanied by a preset low-pass filter^48^,^49^, while KKFs perform Bayesian smoothing using a Markovian update rule that is only learned from the kinematic variables^23^,^28^,^36^. Neither of these approaches smooth the neural activity using properties of the neural activity. For example, the KKF dynamical update rule or OLE low-pass filters may smooth on time-scales that mismatch the time-scales at which the neural activity is informative of kinematics. If the time-constants are too long, then significant lag may be introduced into the system. This work suggests smoothing of the neural data should instead be performed using parameters learned from the neural population responses. That is, given that neural dynamics are informative of the evolution of neural activity through time, incorporating these dynamics (which model the time-constants and rotatory characteristics of the neural population responses) can lead to improved filtering and denoising of neural population responses on single-trials. This study shows that this approach to smoothing the neural activity results in better performance than techniques that do not model neural dynamics. Future work may also assess the extent to which it is beneficial to learn the dynamics of the neural population responses in conjunction with kinematic dynamical update rules.

Interestingly, the NDF achieved similar performance to the state-of-the-art ReFIT-KF^28^ on a similar task with the same monkeys and arrays^34^,^35^. A major reason why this is so is because the NDF is capable of decoding a large dynamic range of velocities, such that it is not only able to move quickly to targets, but is also able to stop more precisely than other decoders, much like the ReFIT-KF. However, the NDF utilizes a different mechanism to achieve this (denoising via dynamical estimation in neural state space) than the ReFIT-KF algorithm (kinematic intention estimation^28^,^50^ and closed-loop adaptation). Future work may assess the extent to which these two different approaches can be combined, since the kinematic intention estimation innovations of the ReFIT-KF are complementary to neural dynamical estimation.

These results contribute to understanding motor cortex as a dynamical system by characterizing the dynamics of single-trial motor cortical activity, demonstrating forward predictivity of neural dynamics on single-trials, and demonstrating an application whereby using neural dynamics can increase BMI performance. This work further supports the idea that motor cortex is not merely an input driven cortical area, but may have its own internal drive. This internal drive describes how previously observed neural activity is informative of yet-to-be observed neural activity. These dynamics can capture salient features of the neural population responses and can beneficially augment single-trial neural state estimation. In one application domain, brain-machine interfaces, where decodes are performed on noisy single trials, incorporating neural dynamics can substantially increase decoder performance. This is a prime example where neuroscientific findings can beneficially inform the design of brain-machine interfaces.

## Methods

### Experimental setup

All surgical and animal care procedures were performed in accordance with National Institutes of Health guidelines and were approved by the Stanford University Institutional Animal Care and Use Committee. Experiments were conducted with adult male rhesus macaques (J and L) implanted with 96 electrode Utah arrays (Blackrock Microsystems Inc., Salt Lake City, UT) using standard neurosurgical techniques. Electrode arrays were implanted in dorsal premotor cortex (PMd) and primary motor cortex (M1) as visually estimated from local anatomical landmarks. Monkey J had two arrays, one in M1 and one in PMd, while Monkey L had one array implanted on the M1/PMd border. Monkey J was 11 years old, and Monkey L was 16-17 years old during experimentation, with arrays implanted 40 and 60 months before experimentation. The monkeys made point-to-point reaches in a 2D plane with a virtual cursor controlled by the contralateral arm or by a BMI. The experimental setup has been previously described^28^,^29^,^30^ and an illustration of the experimental setup is shown in Supplementary Fig. 1. The virtual cursor and targets were presented in a three-dimensional (3D) environment (MSMS, MDDF, USC, Los Angeles, CA). Hand position data were measured with an infrared reflective bead tracking system (Polaris, Northern Digital, Ontario, Canada). Spike counts were collected by applying a single threshold, set to −4.5 × root-mean-square of the spike voltage per neural channel. The raw neural observations used for analyses were binned threshold crossings counted in non-overlapping 15-ms bins. Behavioural control and neural decode were run on separate PCs using Simulink/xPC platform (Mathworks, Natick, MA) with communication latencies of 3 ms. This enabled millisecond timing precision for all computations. Neural data were initially processed by the Cerebus recording system (Blackrock Microsystems Inc., Salt Lake City, UT) and were available to the behavioural control system within 5±1 ms. Visual presentation was provided via two LCD monitors with refresh rates at 120 Hz, yielding frame updates of 7±4 ms. Two mirrors visually fused the displays into a single 3D percept for the user, creating a Wheatstone stereograph. All tasks presented in this study were restricted to a two-dimensional plane. Because this study deals with the dynamics of reaching, we used an animal model where the monkey was free to move the contralateral arm. In the context of BMI, we believe this animal model more closely mimics the neural state of a human subject that would be employing a BMI in a clinical study than a monkey with both arms restrained^45^. However, as noted in the Discussion, we believe that it is important to study the dynamics of imagined movements.

### Tasks

For all experiments conducted in this work, two tasks were utilized. The first was a center-out-and-back reaching task, which was used as a training set for each decoder. The second was a grid task, which was used to evaluate the performance of each decoder. The grid task was used as the evaluation task because it is a selection task that can convey information in a clinically relevant way. The grid task allows the computation of an achieved bitrate which quantifies the rate at which the BMI can communicate information^35^.

#### Center-out-and-back task

In the center-out-and-back task, eight targets were placed with uniform spacing on the circumference of a 12-cm radius circle. The subject was required to acquire the center target, followed by one of the eight (randomly chosen) radial targets. The subject was given 2 s to acquire each prompted target. After successful acquisition of a radial target, or following the failure to acquire any target, the subject was prompted to acquire the center target. Each target had a 4 × 4 cm acceptance window centered around the target. For every target selection, the subject had to hold the cursor within the acceptance window of the target for 500 contiguous milliseconds. Training sets for the decoder were comprised of 500 successful trials during which the subject would repeatedly acquire peripheral and center targets. When necessary, a variant of the center-out-and-back task, with eight targets placed on the circumference of an 8-cm radius circle, was used for cross-validating results.

#### Grid task

The grid task is a generalization task previously used and described by our group which allows for the calculation of an achieved communication rate^34^. In this study, the grid task comprised a 6 × 6 array of targets, each with a 4 × 4 cm acceptance window. The targets were tiled end-to-end contiguously to create a workspace that was 24 × 24 cm. This grid of targets mimics a keyboard task^34^,^35^ where the subject can select any of 36 targets at any time by dwelling in the acceptance window of a target for 450 ms. Because any target can be selected at any time, a correct target selection conveys information; for example, the targets could be alphanumeric characters or symbols from a keyboard. To evaluate performance, the subject had to acquire 1 prompted target out of the potential 36 targets. Although only one target was prompted, every target was selectable if dwelt on for 450 ms. The subject was given 5 s to acquire the prompted target; if no target was selected in 5 s, no target selection would be made. Following target selection, a ‘lock-out' time of 200 ms was enforced, during which the task would continue to run (that is, the next target would be prompted) but dwell time was not counted; this was done to account for the reaction time of the monkey. Targets were randomly chosen according to a uniform distribution, and therefore, the information conveyed per target selection is log_2_(36) bits. To be conservative in the estimation of achieved bitrate, we compensated every incorrect selection with a correct selection, much like an incorrect selection on a keyboard must be corrected by pressing the delete key. Therefore, the information conveyed on the grid task is calculated by considering the net number of correctly selected targets. Hence, performing the task at a success rate of 50% results in a bitrate of 0 bits per second (b.p.s.), so that no information is conveyed through the task. We calculated an achieved information rate (bitrate) by dividing the amount of information conveyed during target acquisition by the time taken to acquire the targets. Therefore, if in *T* seconds, *c* correct selections were made, while ℓ incorrect selections were made, the bitrate was calculated to be:


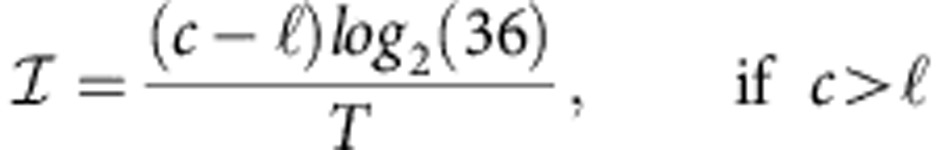


and 
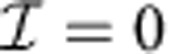
 if *c*≤ℓ. This is the achieved bitrate of the decoder on the grid task. To evaluate the performance of a decoder, the monkey performed the grid task in blocks of approximately 100 trials, from which a bitrate across those trials was calculated. Because the monkey's motivation degrades as the experiment progresses, we evaluated the NDF as the last decoder in each block to ensure that benefits were not due to degrading motivation; the order of decoders tested in each block was therefore deterministic. Each block was run so that decoders were effectively tested in an A–B–A–B–A–... manner (A–B–C–A–B–C–A–... for three decoders, and so forth). The bitrates in each block were paired for statistical testing. We compared the mean performance of each decoder by calculating the mean bitrate across all experimental blocks. Because a positive bitrate, 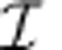, can be approximated as (a scaled) sum of 100 binary random variables, which take on values 1 or −1, the distribution of their sum (that is, the bitrate within a block) will approach a normal distribution as more trials are collected. We used the paired *t*-test to test a difference in the means of the bitrates. Collecting the bitrate estimates in a blocked setting, and across experimental days, better justified an assumption of independence. We collected at least 1,000 trials (>10 samples) to determine the mean bitrate, based both on experimental constraints and to better justify an assumption of normality in the mean bitrate. Nevertheless, although we used the parametric *t*-test in this study, we also performed a Wilcoxon signed-rank test on the paired bitrate differences under the null hypothesis that the median bitrate difference is zero, as well as a Wilcoxon–Mann–Whitney rank sum test for a difference in bitrate distributions. All bitrate differences were significant under these non-parametric tests (*P*<0.05).

### Contribution from the dynamical versus innovations process

As in Fig. 1f, we sought to calculate the contribution of the dynamics process versus the innovations process (taking into account the neural observations) in estimating the next neural state. By the definition of the linear dynamical system used in this work, we have that 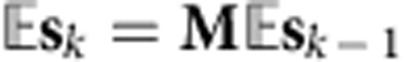, where 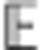 denotes expectation. That is, the progression of the neural state is on average given by the dynamics update. The innovations process, which is a process with zero mean, updates the estimate of the neural state, 
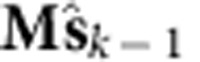
 by using the observed neural data, **y**_*k*_. The innovations are what cannot be explained in the neural data by our observation process, i.e., 
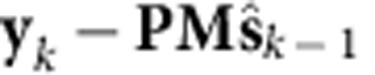
. These innovations are projected by the Kalman filter gain, 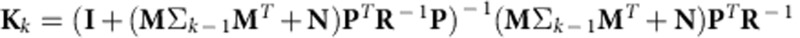, where 
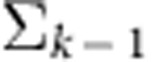
 is the covariance of the estimate 
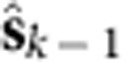
. Hence, the Kalman filter estimate of the neural state at time *k* is


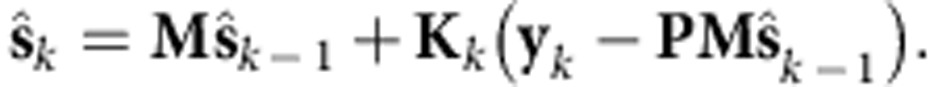


We calculated the contribution of both the dynamics process and the innovations process in predicting the next state from the previous state, that is, 
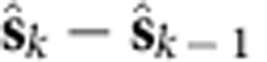
. The contribution from the dynamics process is 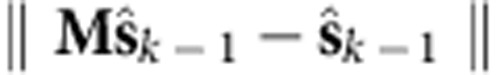, while the contribution from the innovations process is 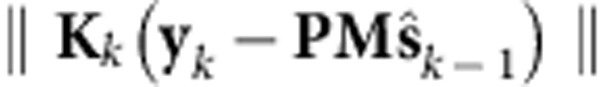. Therefore, to calculate the contribution from the dynamical state update process versus the innovations process, we calculated the ratio:


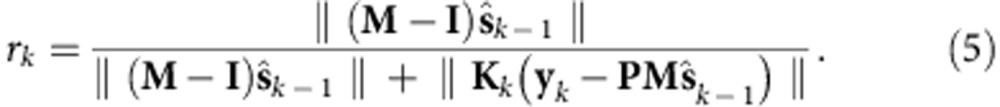


(An intuition for this ratio is that 
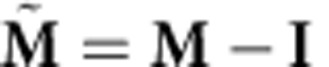
 is the first order approximation to the continuous dynamics matrix, 
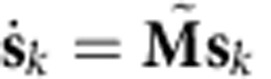
, where 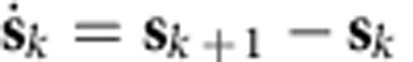. Thus, this ratio calculates the relative contribution to the neural state velocity, as implied by Fig. 1f).

We measured the average of this ratio, 
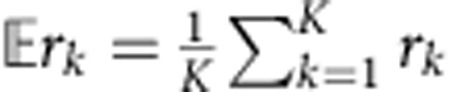
 where *K* is the number of neural observations (i.e., number of observations across time). Across 13 experimental days in Monkey J, where a new LDS was learned on each experimental day, we found the average of this ratio, 
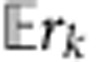
, to be 0.37±0.02 (mean±s.d.), while for 16 experimental days in Monkey L, we found it to be 0.49±0.04.

### Decode algorithms

We utilize the following abbreviations for decoders: the neural dynamical filter (NDF), the optimal linear estimator (OLE), the Wiener filter (WF), and the kinematic-state Kalman filter (KKF). In all decoders, the decoded kinematics are the 2D position (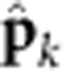) and 2D velocity (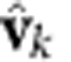) of a computer cursor. Neural spikes were counted in non-overlapping 15-ms bins, and were used as the observations for all decode algorithms. Our choice of bin width is informed by a previous result in online BMI experiments, which demonstrated that smaller bin widths lead to increased performance^30^. Given that the decoded position and velocity of the cursor at time *k* were 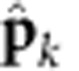 and 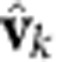 respectively, the decoded position shown to the subject, **p**_*k*_, was calculated as:


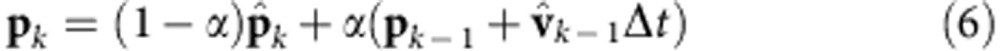


with *α*=0.975 and Δ*t* being the bin width of the decoder. This indicates that the final decoded position is a weighted sum, with 2.5% contribution from the decoded position, and 97.5% contribution from the integrated velocity. The small position contribution in part stabilizes the position of the decoder in the workspace^29^. Other work has noted the importance of taking into account the position contribution of the signal^28^.

All decoders were trained using data collected while a subject made reaches on a center-out-and-back task for 500 successful trials. Although the decoders were trained using data collected while the subject performed a center-out-and-back task, all decoders were evaluated on the grid task.

*Neural dynamical filter*. To learn a NDF, we modelled the following latent state linear dynamical system:


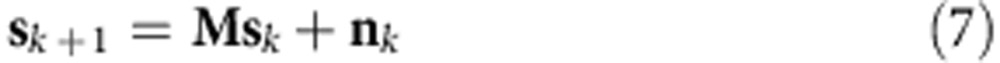



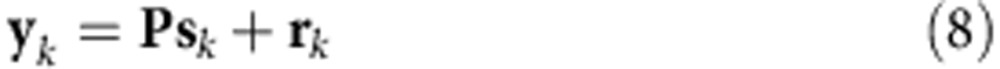


where **n**_*k*_ and **r**_*k*_ are zero mean Gaussian noise terms with diagonal covariance matrices **N** and **R**, respectively. We learned this latent state linear dynamical system in an unsupervised fashion from the sequence of observed neural activity. The time series of neural observations {**y**_*k*_}_*k*=1,2…,*K*_ were treated as the observed output of a latent state LDS. We did not perform any preprocessing steps on the binned spike counts, **y**_*k*_. We performed expectation maximization (EM) to learn the parameters (**M**, **P**, **N**, **R**) of the LDS, as described in a previous report^24^. Briefly, the E-step involves computing the expected joint-log likelihood of the neural state and the neural observations, which can be deduced from the graph structure of Fig. 5a:


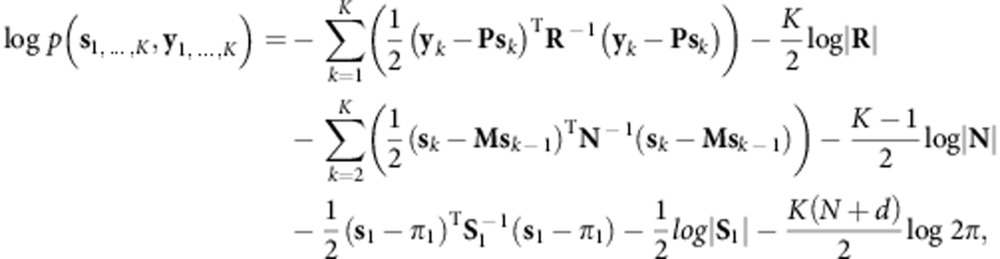


where 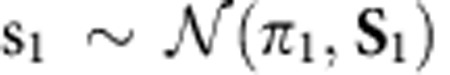 and *N* and *d* are the number of channels and the dimensionality of the latent state, respectively. The joint log-likelihood, given all parameters, can be computed via Kalman smoothing. The M-step then involves maximizing the parameters (**M**, **P**, **N**, **R**, π_1_, **S**_1_) with respect to the joint log-likelihood. We note that while we computed π_1_ and **S**_1_, they were of no practical consequence when running in closed-loop after several seconds. The E-step and M-step alternated to increase the log-likelihood of the observed data. More details can be found in the report by Ghahramani and Hinton^24^. When performing EM, we utilized an approximation in the E-step: we assumed that the Kalman smoothing parameters remained constant after convergence of the estimated state covariance matrix within reasonable tolerance. In the offline analyses of this study, the EM algorithm was initialized with factor analysis. In online prosthetics experiments, we also learned dynamical systems where the EM algorithm was initialized using previously learned dynamical systems. Initialization from a previously learned LDS decreased the convergence time and sometimes resulted in more optimal LDS' (since EM is subject to local optima). We briefly evaluated the performance of NDF algorithms using each of the learned dynamical systems, and chose the one with the highest performance.

After learning the parameters of the latent state dynamical system via EM, we used the steady-state form of the Kalman filter to estimate the neural state, 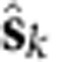, at each point in time from the sequence of neural observations, **y**_*k*_, in the training data. It was reasonable to use the computationally efficient steady-state form of the Kalman filter, since convergence to steady-state occured on the order of seconds. We thus had a sequence of decoded neural states, 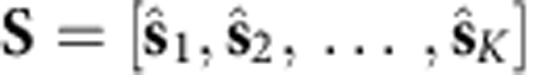 and a corresponding sequence of observed training set kinematics, 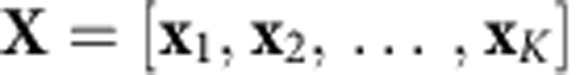, where **x**_*k*_ contains the position and velocity of the hand-controlled cursor at time *k*. We then found the matrix **L**_*s*_, which minimizes the mean squared error, ||**X**–**L**_*s*_[**S**; **1**]||^2^, where **1** refers to a row of 1's appended to the bottom of **S** to allow for a bias to be learned. After defining **S**_*b*_=[**S**; **1**], the solution is 
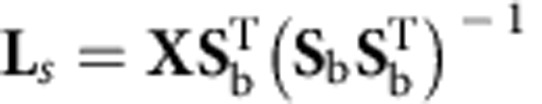
.

We note two related offline BMI studies; the study by Wu *et al*.^40^ utilized a latent state, while the study by Truccolo *et al*.^41^ modelled temporal interactions across the neurons. In the study by Wu and colleagues, a latent state model was learned in conjunction with the observed kinematics, so that the latent dynamical process is coupled to the kinematics. Interestingly, it was found that with this model, the parameters could not be identified with EM when the hidden state dimensionality was >3, which suggests that it does not adequately capture the relatively higher-dimensional neural dynamics^10^. In the study by Truccolo and colleagues, it was found that the interactions across neurons was only significant for 3–5 ms, which are of far shorter time scales than those used in this study.

*Optimal linear estimator*. The OLE^31^ was fit by solving the least-squares regression problem between the sequence of observed kinematics in the training set, **X**, and the corresponding sequence of observed neural data, **Y**=[**y**_1_,**y**_2_,…,**y**_*K*_]. Analogous to the NDF case, we solved for the matrix **L**_*y*_ minimizing the mean squared error ||**X**–**L**_*y*_[**Y**; **1**]||^2^. We then defined **Y**_b_=[**Y**; **1**], so that a row of 1's was appended to the bottom of the matrix to account for a bias term. The solution is 
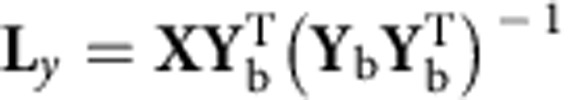
. To allow for the sequence of neural data to have smoothness, we also convolved every row of **Y** with causal Gaussian kernels having standard deviations ranging from 25–200 ms.

*Wiener filter*. The WF incorporates neural history into the regression problem by finding the optimal coefficients for historical neural data. The Wiener–Kolmogorov filtering approach finds the optimal matrices, **L**_0_, **L**_1_,…, **L**_*p*−1_ such that 
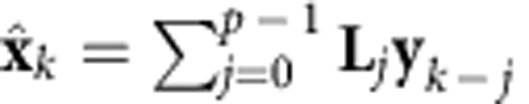
, where the difference between the decoded and observed kinematics is minimized in the least-squares sense. Hence, the WF operates on a history of neural data of length *p*Δ*t*, where Δ*t* is the bin size in which spikes were counted. To fit the WF, we first define **X**_[*i*:*j*]_=[**x**_*i*_ **x**_*i*+1_…**x**_*j*_] for *i*<*j*, and the following matrix:


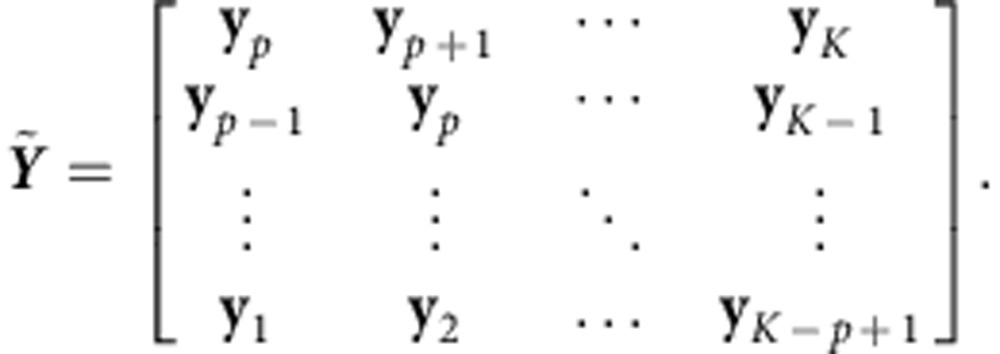


where *p* is a parameter denoting the amount of history used in the decoder, and *K* is the total number of bins observed. By defining **L**_*W*_=[**L**_0_ **L**_1_…**L**_*p*−1_ ***b***_*WF*_], the horizontal concatenation of the matrices **L**_*j*_ for *j*=0, 1,…,*p*−1 (and a bias term), we could solve for **L**_*W*_ such that the error metric 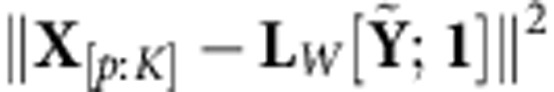 was minimized. After defining 
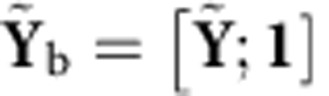
, the solution is 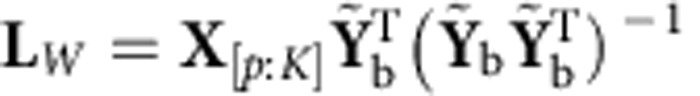. Analogously to least-squares, the term 
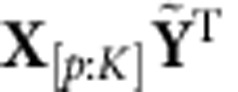
 represents the time-averaged cross-correlations between the kinematics and neural activity up to *p* bins in the past, while the term 
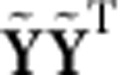
 represents the time-averaged autocorrelations of the neural data up to *p* bins in the past. To avoid overfitting, we also regularized the regression, so that we found 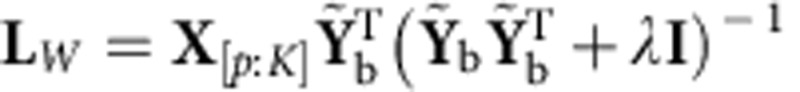. Both parameters *p* and λ were found by optimization in online control, where *p* and λ were swept and the performance of the WF evaluated. We found that the optimal amount of history to use was ∼250 ms for both subjects, although the minimum was shallow in the range of 200–300 ms. This is a significantly smaller history than that used in previous works^33^,^37^,^51^,^52^. We observed a degradation in performance when the history was greater than 500 ms due to a noticeable lack of fine-control in the cursor. This is because assigning significant weight to neural data relatively far into the past will cause the decoder to have significant lag in responding to the subject's changing intention.

*Kinematic-state Kalman filter*. The KKF models the kinematics at time *k*, **x**_*k*_, as the state of a linear dynamical system, while the simultaneously observed neural population spike counts, **y**_*k*_, are the corresponding output of the system. This is represented by the two equations,


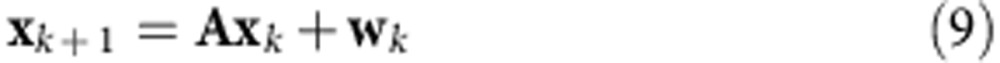



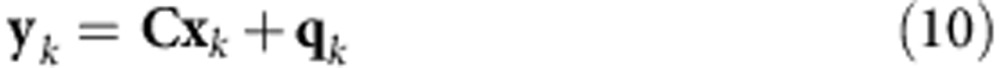


where **w**_*k*_ and **q**_*k*_ are zero mean Gaussian noise terms with covariance matrices **W** and **Q**, respectively. As the sequences {**x**_*k*_}_*k*=1,…,*K*_ and {**y**_*k*_}_*k*=1,…,*K*_ were observed in the training set while **w**_*k*_ and **q**_*k*_ are zero mean terms, **A** and **C** can be learned via least-squares regression: **A** and **C** can be calculated as: 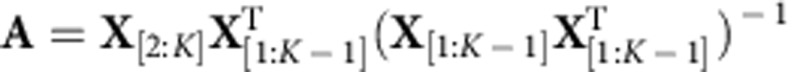 and **C**=**YX**^T^(**XX**^T^)^−1^. After learning **A** and **C**, **W** was calculated as the sample covariance of the residuals **X**_[2:*K*]_–**AX**_[1:*K*−1]_, while **Q** was analogously the sample covariance of the residuals **Y**–**CX**. Given these parameters, and an initial condition (
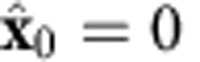
), the Kalman filter is a recursive algorithm which estimates the state at time *k*, 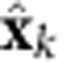, given the previous state estimate, 
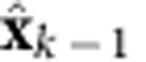
, and the current observation, **y**_*k*_. A strength of this construction is the smoothness in the kinematics afforded by modelling kinematic update laws in the matrix **A**. However, we note that this model does not capture any temporal structure in the neural population activity. While 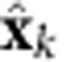 reflects, to an extent, the history of the neural data in that 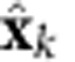 can be written as a linear combination of **y**_*k*_, **y**_*k*−1_,…, **y**_1_, the temporal evolution of 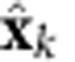 is governed by the linear dynamics of the kinematics, and does not take into account any temporal correlations in the neural data. When presenting decodes to the monkey, we found that a pure velocity Kalman filter performed inferiorly to one where the position is decoded as in equation (6).

## Additional information

**How to cite this article:** Kao, J. C. *et al*. Single-trial dynamics of motor cortex and their applications to brain-machine interfaces. *Nat. Commun.* 6:7759 doi: 10.1038/ncomms8759 (2015).

## Supplementary Material

Supplementary Information Supplementary Figures 1-6 and Supplementary Tables 1-3

Supplementary Movie 1 Demonstrates the performance of NDF on the grid task. The video was reconstructed from kinematic data of the neural prosthesis cursor and plays in real-time, with timings identical to the live task viewed through a 30 fps framerate. The targets highlighted in green are the cued targets the monkey must acquire; when the target is blue, it indicates that the cursor was brought within the acceptance window of the target. In this video, the bitrate achieved by the NDF is 4.2 bps.
