# Supplementary material for: Single-trial dynamics of motor cortex and their applications to brain-machine interfaces
Source: Nat Commun. 2015 Jul 29;6:7759. doi: 10.1038/ncomms8759 (PMC4532790; doi:10.1038/ncomms8759)
Supplement: Supplementary Information — Supplementary Figures 1-6 and Supplementary Tables 1-3 [file ncomms8759-s1.pdf]

Single-trial dynamics of motor cortex and their applications to brain-machine interfaces

Jonathan C Kao

Paul Nuyujukian

Stephen I Ryu

Mark M Churchland

John P Cunningham

Krishna V Shenoy

Supplement

Supplementary Figures

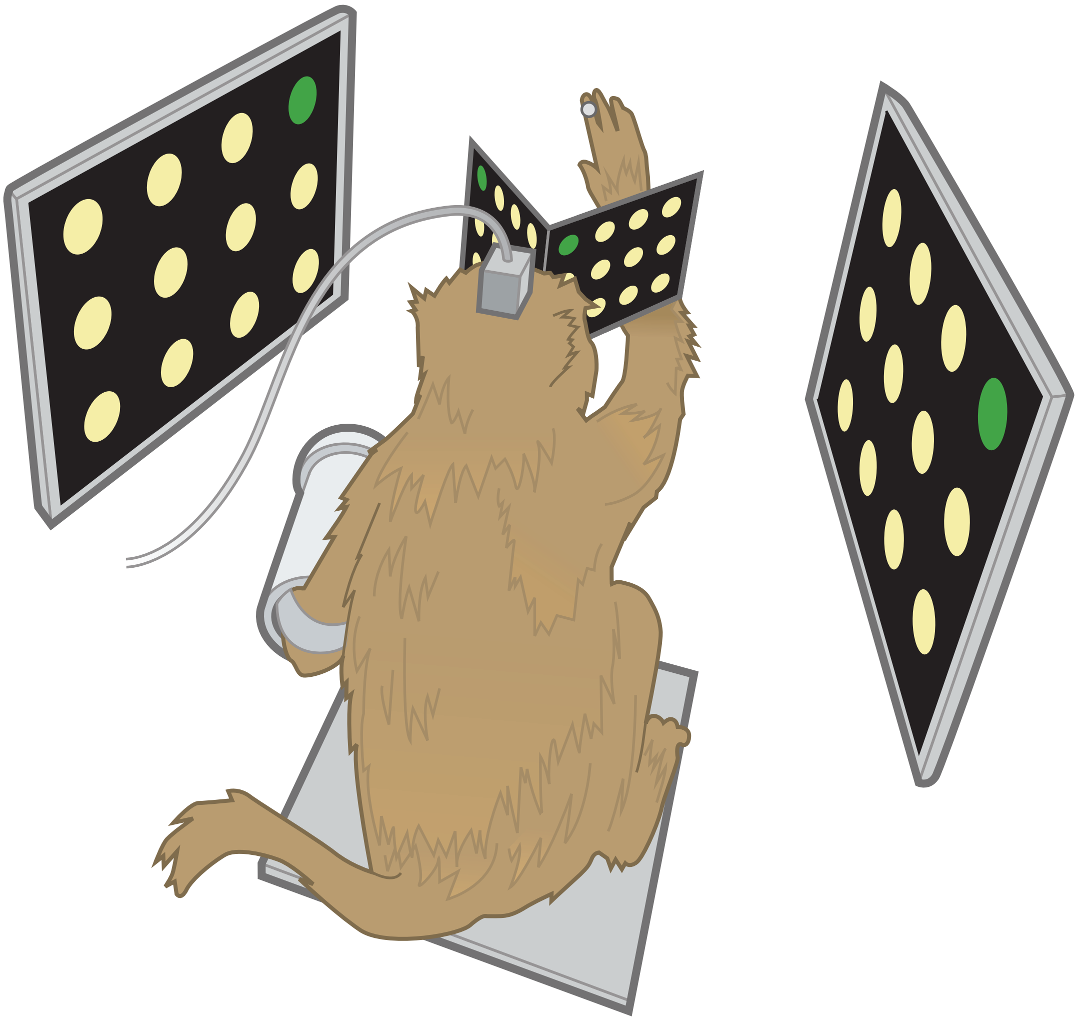

Supplementary Figure 1: Experimental setup. A monkey was positioned in front of two mirrors reflecting the content of two monitors, as in a Wheatstone stereograph[1]. Thus, the monkey perceived the content of the monitor in the space in front of him. The monkey acquired the presented targets while neural activity was recorded, and hand movements were tracked using an infrared bead. All events were tracked with millisecond level precision. Two tasks were used (further described in the Methods). This figure shows a grid task with 12 targets, although we note that the grid task used to evaluate performance in this study was a 6×6 grid of targets.

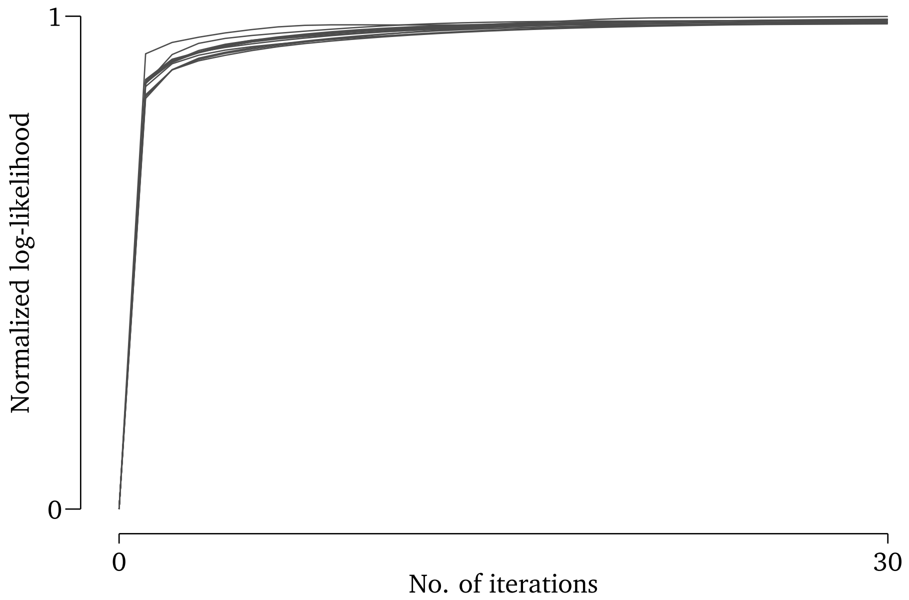

Supplementary Figure 2: Cross-validated log-likelihood. The cross-validated log-likelihoods (normalized to the range $[0, 1]$) are shown for seven LDS's in Monkey J and five LDS's in Monkey L across 30 trials of the EM algorithm. The increase in likelihood until a plateau shows that the LDS is not merely fitting noise in the neural population responses, but is capturing underlying structure in the activity.

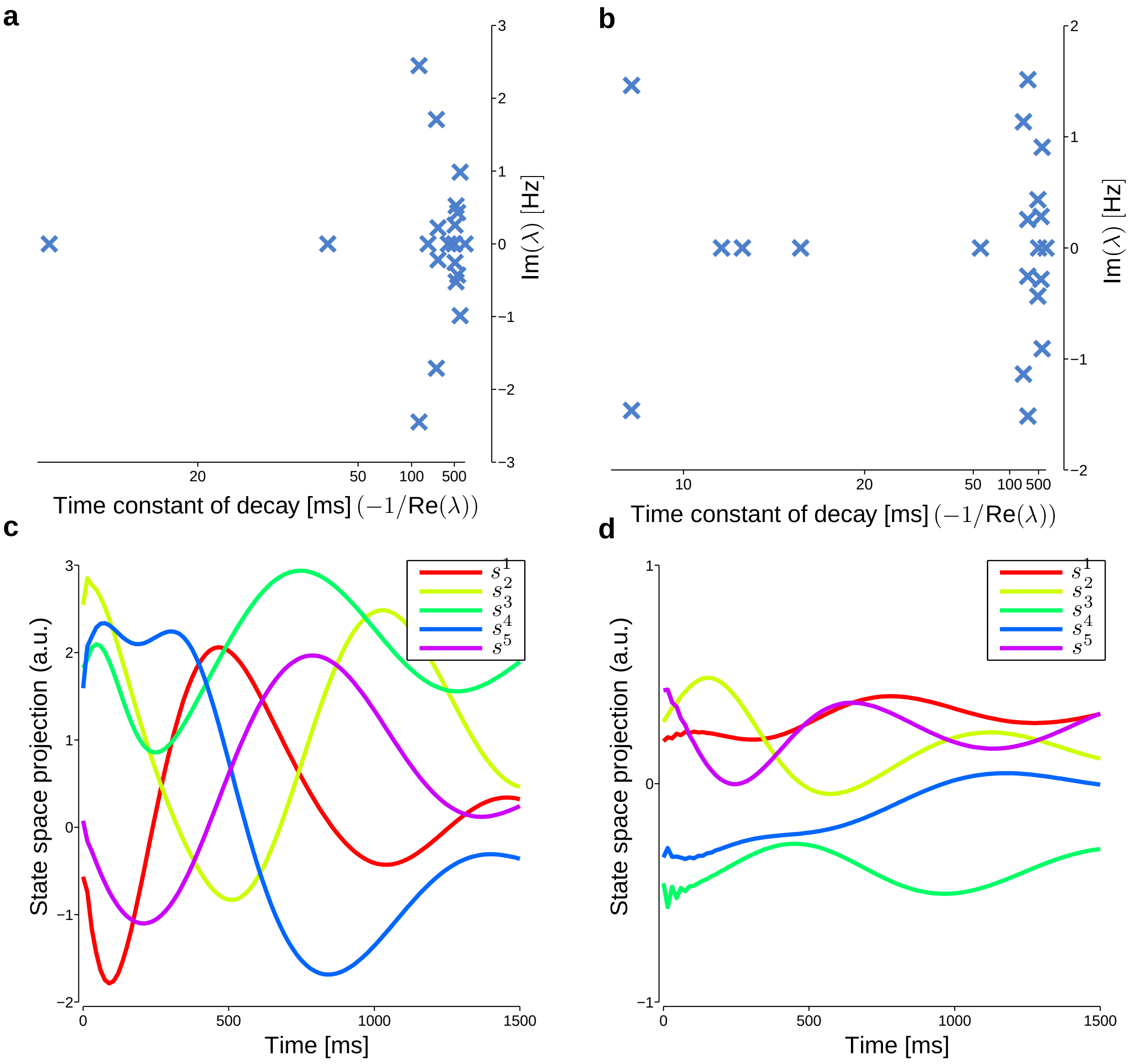

Supplementary Figure 3: Eigenvalue spectrum of the neural dynamics. **(a)** Example spectra of the first-order Euler approximation of the continuous dynamics matrix, $\tilde{\mathbf{M}} = \mathbf{M} - \mathbf{I}$ for Monkeys J & L respectively. The time constants of decay for most eigenvalues are hundreds of milliseconds, while the frequencies used are up to 2.5 Hz. As all eigenvalues of $\tilde{\mathbf{M}}$ have real part less than 0, or equivalently, the eigenvalues of $\mathbf{M}$ have real part less than 1, the eigenvalues are all stable. We also found that the eigenvalues are all distinct. A more detailed view of the time constants and frequency response across all learned dynamical systems is shown in Supplementary Fig 4. **(b)** Same as (a) but for Monkey L. **(c)** The neural state (Monkey J) is initialized to a point in state-space and the expected trajectory is computed, via $\mathbb{E}\mathbf{s}_k = \mathbf{M}\mathbb{E}\mathbf{s}_{k-1}$. In the legend, s^i denotes the ith dimension of the neural state. The trajectories demonstrate both exponential decay and oscillations. **(d)** Same as (c) but for Monkey L.

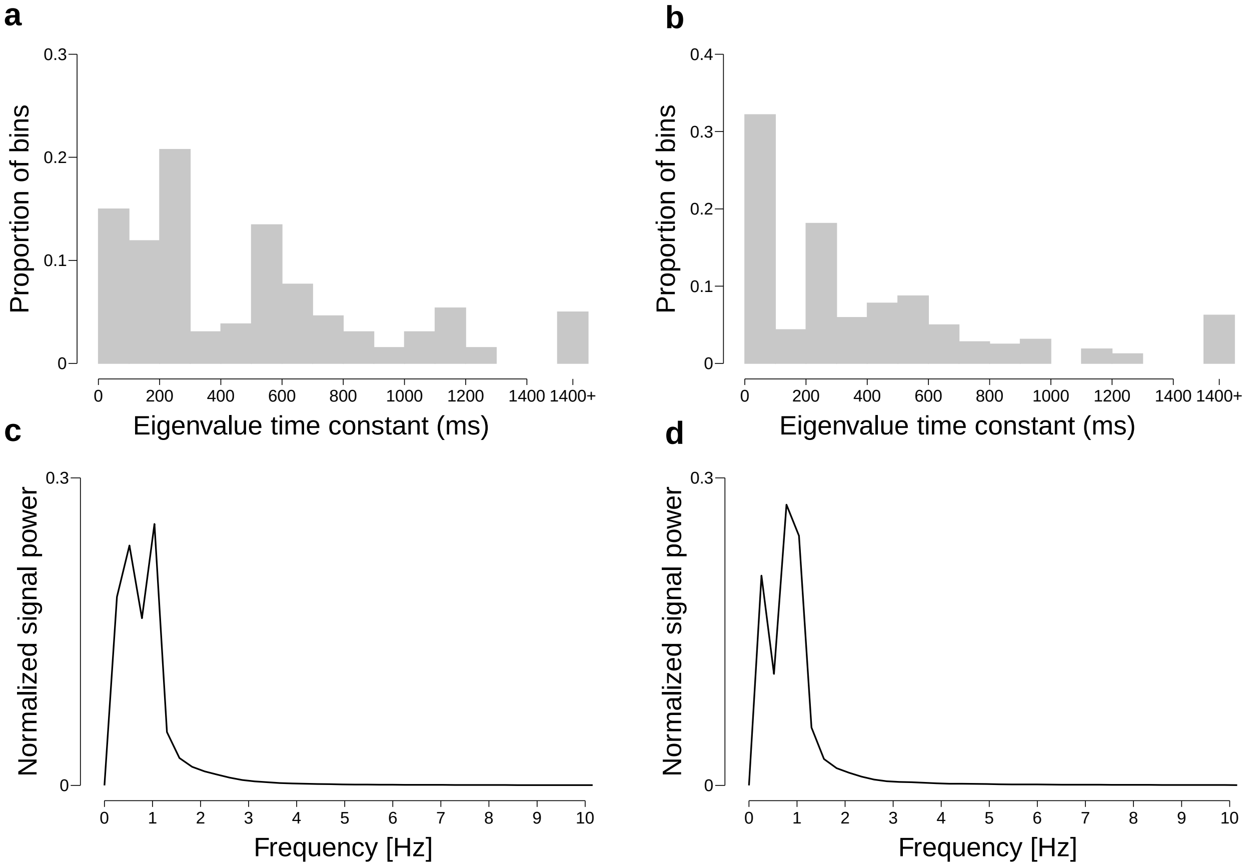

Supplementary Figure 4: Dynamical system eigenvalue characteristics. **(a)** The histogram of the time constants learned from the neural population responses across all experimental sessions are shown for Monkey J. The time constants are $-1/\text{Re}\{\lambda\}$ of the eigenvalues for the continuous dynamics matrix, $\tilde{\mathbf{M}}$. **(b)** Same as (a) but for Monkey L. **(c)** This frequency response captures the power in each frequency when the neural state evolves according to the dynamical model. The neural state is initialized to the initial state found by EM and propagates according to its dynamics (a pseudo-impulse response). The Fourier transform is then taken for each dimension of the neural state, and averaged across all dimensions, and subsequently all linear dynamical systems. In Monkey J, we found that the dominant frequency was 1.04 Hz (corresponding to approximately one oscillation per reach) with a subharmonic at 0.052 Hz, These results are consistent with previously reported values[2] on simple reaching tasks. **(d)** Same as (c) but for Monkey L. For Monkey L, we found that the most powerful frequency was at 0.78 Hz with a subharmonic at 0.26 Hz.

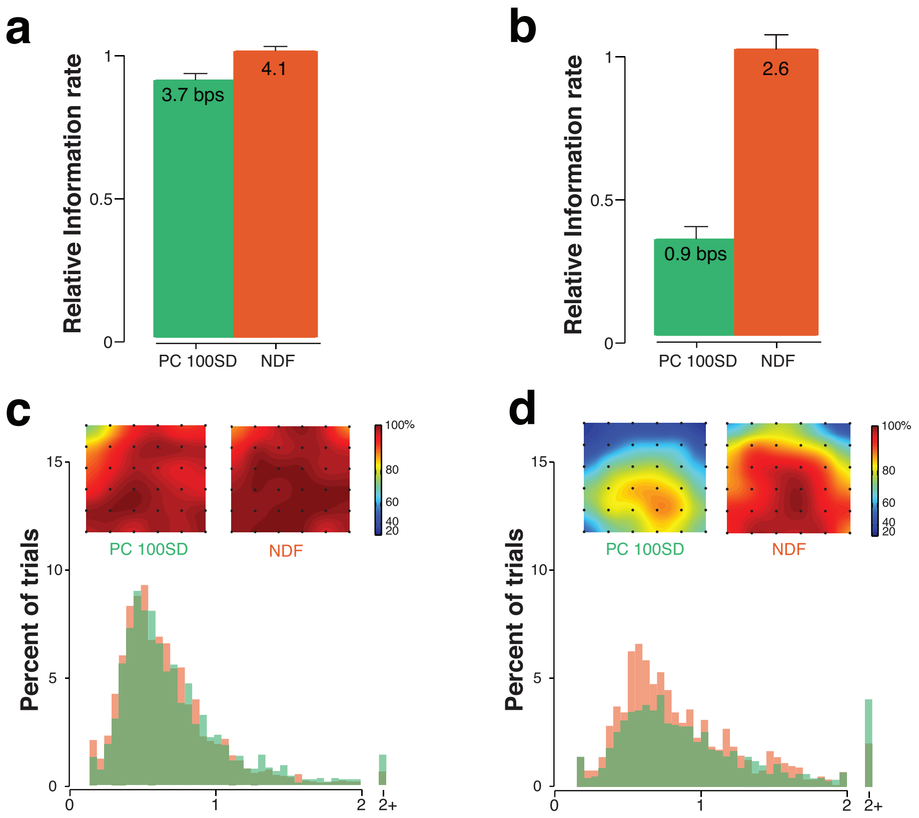

Supplementary Figure 5: NDF outperforms a PCA decoder with smoothing. We performed an additional closed-loop experiment where we compared the NDF to a PCA decoder with smoothing ("PC-smooth"). The dimensionality of the neural observations, $\mathbf{y}_k$, was reduced to 20-dimensions using PCA. We performed PCA similar to previous studies using neural activity[2–4] by finding the principal components on condition-averaged data since PCA is susceptible to noise[5]. However, we did not perform variance normalization on the observations[6], $\mathbf{y}_k$, so as to provide a fair comparison to the LDS where the neural observations were similarly not variance normalized. We smoothed the neural observations before performing dimensionality reduction, which is equivalent to smoothing the components in time due to the linearity of convolution. We found that even when the dimensionality was equalized and the components smoothed in time, the NDF performed superiorly to the PC-smooth decoder (panels a,b, bars are mean and error bars are s.e.m.). The NDF demonstrated an 11% and 198% improvement over PC-smooth in Monkeys J and L, respectively ($p < 0.01$, paired t-test, bitrates estimated from 3792 total online trials for Monkey J and 2923 trials for Monkey L). The poorer performance in Monkey L may be partially attributable to the presence of high firing rate (and thus high variance) channels that were not modulated for the task, which may have subsequently skewed the PCs. The average success rates were 95% and 97% for PC-smooth and NDF in Monkey J, and 62% and 85% in Monkey L. The average acquire times were 747 ms and 696 ms for PC-smooth and NDF in Monkey J, and 989 ms and 891 ms in Monkey L. This demonstrates that the NDF outperforms a decoder that reduces dimensionality and smooths the components in time.

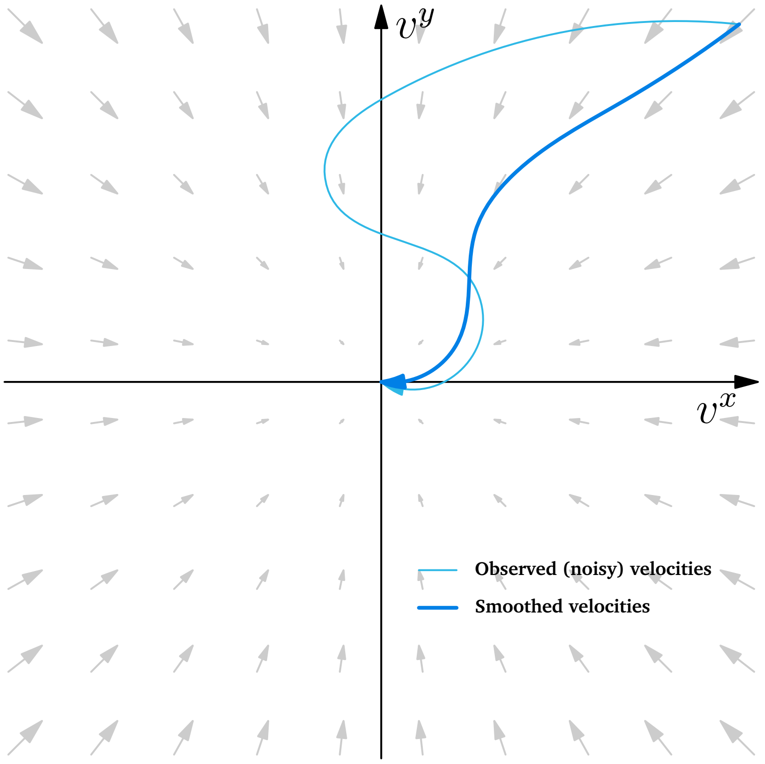

Supplementary Figure 6: Velocity smoothing in BMIs. This figure illustrates an example, demonstrating how velocity Kalman filtering can help smooth decoded kinematics. In this example, the subject is in the middle of moving a prosthetic cursor in the positive x and y directions at a high velocity. The subject then slows down the cursor. While slowing down the cursor, the neural observations (due to noise) may have immediately decoded cursor movement to the left (i.e., v^x negative). However, by incorporating smoothness in the velocities, the decoded cursor movement continues to go in the approximately same direction and decays to zero velocity.

Supplementary Tables

	Hand (J)	Neural observation (J)	Neural state (J)	Smoothed neural (J)	Hand (L)	Neural observation (L)	Neural state (L)	Smoothed neural (L)
Average velocity (hold epoch) (cm/s)	4.7	6.1	6.7	7.6	6.7	6.0	7.8	8.5
Average velocity (reach epoch) (cm/s)	13.7	10.5	15.3	15.5	15.3	8.7	15.6	14.6
Dynamic Range (cm/s)	**9**	4.4	**8.6**	7.9	**8.6**	2.7	**7.8**	6.1

Supplementary Table 1: Smoothing control for decoding single-trial velocities during reaching and holding. This table summarizes the average hold and reach velocities decoded on cross-validated single-trials when the input signal is the high-dimensional neural data ($\mathbf{y}_k$, OLE, see Methods) versus the dynamical neural state (s_k, NDF, see Methods). Because neural activity is highly noisy, we performed a control of the results in Fig 3 where neural activity was smoothed by a causal Gaussian kernel. We smoothed the neural data until the decoded reach velocities were not significantly different (or slightly less) than the neural state decoded reach velocities. The first block are results for Monkey J while the second block are results from Monkey L. For both monkeys, the causal Gaussian kernel had a standard deviation of 50 ms. The first three columns (comparing hand, neural observation and neural state) is the same data shown in Fig 3. In the fourth column (smoothed neural) the same analysis is done, but with smoothing on the neural data such that it can decode quick reach velocities. We found that in both monkeys, when the average reach velocity from the smoothed neural data was not significantly different (Monkey J) or less than (Monkey L) the neural state decoded average reach velocity, that the average hold velocity increased significantly. Moreover, it increased to the point where the average hold velocity decoded by the smoothed neural data was significantly higher than that decoded by the dynamical neural state ($p < 0.01$, both monkeys). Therefore, even with smoothing, the neural data was not able to achieve a dynamic range of velocities as high as that of the dynamical neural state. Hence, decoding with the dynamical neural state not only allows the decoding of high velocities, but also appropriately low velocities (for e.g., fine control).

	OLE	OLE, 25SD	OLE, 50SD	OLE, 100SD	OLE, 200SD	NDF	KKF	NDF	WF	NDF
Bitrate (bps)	0.2	2.7	3.2	3.0	2.6	**4.16**	2.62	**3.83**	3.50	**4.06**
Success rate (%)	41	84	90	89	86	**98**	92	**97**	92	**97**
Acq Time (ms)	774	756	801	825	950	**692**	1185	**774**	731	**697**
Trial Counts	1288	2976	2979	2985	3029	2988	2244	2256	2291	2270

Supplementary Table 2: For Monkey J, the mean statistics (averaged across experimental blocks) is shown for all decoders. These statistics are the bitrate, success rate of target acquisition, and the average time taken to acquire each target for each decoder in closed-loop control. We note that the success rate, which is the proportion of correct selections, is expected to be lower than when the subject has to only acquire a prompted target, such as in a center-out-and-back task. This is because in the grid task, incorrect trials include those trials where the cursor dwelled on any uncued target in the workspace, e.g., when the cursor is en route to the correct target but moves too slowly, as shown in Supplementary Movie 1. The target acquire time does not include the constant 450 ms hold time, and incorrect trials are not included in this average. A normalized acquire time (where the target acquire time is divided by the distance between successive targets) resulted in the same trends, as trial counts were large enough such that the average distance between targets was approximately the same across all collected experimental blocks. The performance differences for the NDF decoder across experimental comparisons (OLE vs NDF, KKF vs NDF, WF vs NDF) can be attributed to several factors, including monkey motivation (where performance tended to be poorer if the NDF was used in closed-loop control following a poor decoder such as the KKF), the quality of the neural dynamics modeled that day, and performance differences over weeks. However, the data collected within each experimental comparison is paired. The top performing decoder in each category is bolded. The total number of closed-loop BMI trials for performance quantification across all experimental comparisons is $25,306$ trials.

	OLE	OLE, 25SD	OLE, 50SD	OLE, 100SD	OLE, 200SD	NDF	KKF	NDF	WF	NDF
Bitrate (bps)	0	0.05	0.51	1.28	1.23	**2.34**	1.42	**2.29**	2.65	**2.99**
Success rate (%)	N/A	31	54	69	70	**85**	74	**82**	85	**89**
Avg Acq Time (ms)	N/A	1140	1102	1121	1345	**1051**	1327	**984**	872	**860**
Trial Counts	110	502	1767	2024	2031	2138	1831	1852	2136	2160

Supplementary Table 3: Same as Supplementary Table 2, but for Monkey L. The same trends are shown across all experimental comparisons. The total number of closed-loop BMI trials for performance quantification across all experimental comparisons is $16,551$ trials.

References

[1] Cunningham, J. P., Nuyujukian, P., Gilja, V., Chestek, C. A., Ryu, S. I., and Shenoy, K. V. A closed-loop human simulator for investigating the role of feedback control in brain-machine interfaces. *Journal of Neurophysiology* **105**, 1932–1949 (2011).

[2] Churchland, M. M., Cunningham, J. P., Kaufman, M. T., Foster, J. D., Nuyujukian, P., Ryu, S. I., and Shenoy, K. V. Neural population dynamics during reaching. *Nature* **487**(7405), 51–6, July (2012).

[3] Kaufman, M. T., Churchland, M. M., Ryu, S. I., and Shenoy, K. V. Cortical activity in the null space: permitting preparation without movement. *Nature Neuroscience* **17**(3), 440–8, March (2014).

[4] Ames, K. C., Ryu, S. I., and Shenoy, K. V. Neural dynamics of reaching following incorrect or absent motor preparation. *Neuron* **81**(2), 438–451, January (2014).

[5] Yu, B. M., Cunningham, J. P., Santhanam, G., Ryu, S. I., and Shenoy, K. V. Gaussian-process factor analysis for low-dimensional single-trial analysis of neural population activity. *Journal of Neurophysiology* **102**, 612–635 (2009).

[6] Kao, J. C., Nuyujukian, P., Stavisky, S. D., Ryu, S. I., Ganguli, S., and Shenoy, K. V. Investigating the role of firing-rate normalization and dimensionality reduction in brain-machine interface robustness. In *Proceedings of the 35th Annual Conference of the IEEE EMBS*, volume 2010, 3–7, (2013).
